# Plasma N-terminal containing tau fragments (NTA-tau): a biomarker of tau deposition in Alzheimer’s Disease

**DOI:** 10.1186/s13024-024-00707-x

**Published:** 2024-02-17

**Authors:** Juan Lantero-Rodriguez, Gemma Salvadó, Anniina Snellman, Laia Montoliu-Gaya, Wagner S. Brum, Andrea L. Benedet, Niklas Mattsson-Carlgren, Pontus Tideman, Shorena Janelidze, Sebastian Palmqvist, Erik Stomrud, Nicholas J. Ashton, Henrik Zetterberg, Kaj Blennow, Oskar Hansson

**Affiliations:** 1 Department of Psychiatry and Neurochemistry, Institute of Neuroscience & Physiology, The Sahlgrenska Academy at the University of Gothenburg, House V3/SU, 43180 Mölndal, Sweden; 2 Clinical Memory Research Unit, Department of Clinical Sciences Malmö, Lund University, Lund, Sweden; 3 Turku PET Centre, University of Turku, Turku University Hospital, Turku, Finland; 4 Graduate Program in Biological Sciences: Biochemistry, Universidade Federal Do Rio Grande Do Sul (UFRGS), Porto Alegre, Brazil; 5 Department of Neurology, Skåne University Hospital, Lund, Sweden; 6 Wallenberg Center for Molecular Medicine, Lund University, Lund, Sweden; 7 Memory Clinic, Skåne University Hospital, 20502 Malmö, Sweden; 8 Wallenberg Centre for Molecular and Translational Medicine, University of Gothenburg, Gothenburg, Sweden; 9 Department of Old Age Psychiatry, Maurice Wohl Clinical Neuroscience Institute, King’s College London, London, UK; 10 NIHR Biomedical Research Centre for Mental Health & Biomedical Research Unit for Dementia at South London & Maudsley NHS Foundation, London, UK; 11 Clinical Neurochemistry Laboratory, Sahlgrenska University Hospital, Mölndal, Sweden; 12 Department of Neurodegenerative Disease, Queen Square Institute of Neurology, University College London, London, UK; 13 UK Dementia Research Institute, University College London, London, UK; 14 Hong Kong Center for Neurodegenerative Diseases, Hong Kong, China; 15 Wisconsin Alzheimer’s Disease Research Center, University of Wisconsin School of Medicine and Public Health, University of Wisconsin-Madison, Madison, WI USA

**Keywords:** Tau, NTA, NTA-tau, Plasma, Alzheimer’s disease, Biomarkers, BioFINDER, Tau-PET, Tau pathology

## Abstract

**Background:**

Novel phosphorylated-tau (p-tau) blood biomarkers (e.g., p-tau181, p-tau217 or p-tau231), are highly specific for Alzheimer’s disease (AD), and can track amyloid-β (Aβ) and tau pathology. However, because these biomarkers are strongly associated with the emergence of Aβ pathology, it is difficult to determine the contribution of insoluble tau aggregates to the plasma p-tau signal in blood. Therefore, there remains a need for a biomarker capable of specifically tracking insoluble tau accumulation in brain.

**Methods:**

NTA is a novel ultrasensitive assay targeting N-terminal containing tau fragments (NTA-tau) in cerebrospinal fluid (CSF) and plasma, which is elevated in AD. Using two well-characterized research cohorts (BioFINDER-2, *n* = 1,294, and BioFINDER-1, *n* = 932), we investigated the association between plasma NTA-tau levels and disease progression in AD, including tau accumulation, brain atrophy and cognitive decline.

**Results:**

We demonstrate that plasma NTA-tau increases across the AD *continuum*¸ especially during late stages, and displays a moderate-to-strong association with tau-PET (β = 0.54, *p* < 0.001) in Aβ-positive participants, while weak with Aβ-PET (β = 0.28, *p* < 0.001). Unlike plasma p-tau181, GFAP, NfL and t-tau, tau pathology determined with tau-PET is the most prominent contributor to NTA-tau variance (52.5% of total *R*^2^), while having very low contribution from Aβ pathology measured with CSF Aβ42/40 (4.3%). High baseline NTA-tau levels are predictive of tau-PET accumulation (*R*^2^ = 0.27), steeper atrophy (*R*^2^ ≥ 0.18) and steeper cognitive decline (*R*^2^ ≥ 0.27) in participants within the AD *continuum*. Plasma NTA-tau levels significantly increase over time in Aβ positive cognitively unimpaired (β_std_ = 0.16) and impaired (β_std_ = 0.18) at baseline compared to their Aβ negative counterparts. Finally, longitudinal increases in plasma NTA-tau levels were associated with steeper longitudinal decreases in cortical thickness (*R*^2^ = 0.21) and cognition (*R*^2^ = 0.20).

**Conclusion:**

Our results indicate that plasma NTA-tau levels increase across the AD *continuum*, especially during mid-to-late AD stages, and it is closely associated with in vivo tau tangle deposition in AD and its downstream effects. Moreover, this novel biomarker has potential as a cost-effective and easily accessible tool for monitoring disease progression and cognitive decline in clinical settings, and as an outcome measure in clinical trials which also need to assess the downstream effects of successful Aβ removal.

**Supplementary Information:**

The online version contains supplementary material available at 10.1186/s13024-024-00707-x.

## Introduction

Alzheimer’s disease (AD) is neuropathologically defined by the abnormal accumulation of amyloid-β (Aβ) peptides into extracellular Aβ plaques and intraneuronal fibrillary aggregates comprised of phosphorylated tau protein referred to as neurofibrillary tangles (NFTs) [1, 2]. A definitive diagnosis of AD can only be set based on the post-mortem confirmation of these two lesions [3, 4]. On the other hand, according to the the National Institute of Aging and Alzheimer’s Association (NIA-AA) Research Framework, AD is a biological construct defined in vivo by abnormal biomarkers [5]. This research framework groups fluid and imaging biomarkers into the so-called AT(N) classification: “A”, for biomarkers of aggregated Aβ, including cerebrospinal fluid (CSF) Aβ42, CSF Aβ42/40 ratio and Aβ-PET; “T”, for biomarkers of aggregated tau (NFTs), comprising CSF phosphorylated tau at threonine 181 (p-tau181) and tau-PET; and “(N)” (N biomarkers are not AD specific and therefore appeared in parenthesis), for biomarkers of neurodegeneration or neuronal injury, specifically CSF total-tau (t-tau), MRI and FDG PET [5]. However, CSF and PET biomarkers may be regarded as invasive (requiring a lumbar puncture or injection of radioactive molecules), have low availability and cost-effectiveness, and can only be performed at specialized centers, compromising their widespread implementation in clinical practice. Thus, blood biomarkers provide an opportunity to overcome these limiting factors [6].

A milestone in the field of fluid biomarkers in neurodegenerative diseases has been the characterization of tau protein in CSF and blood [7–14], and consequently, the development of several biomarker assays targeting both phosphorylated and non-phosphorylated variants of this protein [15–18]. Soon after the discovery of p-tau as the main component of NFTs [19, 20], a report confirmed the presence of tau protein in the CSF of AD dementia patients [21]. These early findings paved the way for the later development of several CSF immunoassays targeting various p-tau residues (e.g., p-tau181, p-tau231 and p-tau235) and assays targeting tau species irrespective of the phosphorylation state and/or isoform (t-tau) [22–27]. This was soon followed by the first studies comparing the performance of tau immunoassays [28]. Despite ground-breaking at the time, these early reports were limited in content and scope, thus resulting in the idea that the different p-tau residues and t-tau assays offered similar diagnostic and clinical utility. This led to the subsequent validation of mid-region directed CSF p-tau181 and t-tau for clinical use, which have since demonstrated robust and consistent performance in identifying AD [29]. Consequently, the interest in targeting other p-tau residues as well as other non-phosphorylated tau fragments in CSF faded away for many years. Improvements in mass spectrometry and immunoassay methods eventually led to a regain attention in phosphorylated and non-phosphorylated tau protein as biomarkers. As a result, a remarkable expansion in biomarkers measuring different variants of tau protein both in CSF and blood took place in recent years [15–18]. These novel studies characterizing a large spectrum of fluid tau biomarkers advanced the field by demonstrating that different p-tau residues and non-phosphorylated tau fragments provide distinct advantages reflecting clinically relevant aspects of brain pathophysiology. For example, p-tau231 has been shown to be the first p-tau residue abnormally emerging across the AD *continuum*, a unique feature allowing the earliest confirmation of underlying AD pathophysiological changes [30–33]. On the other hand, p-tau217 has been suggested to be a preferable biomarker for AD diagnosis and monitoring, due to its pronounced fold changes and association with neurodegeneration and cognitive decline [31, 33–35]. Regarding t-tau, measurements in blood are more challenging than in CSF, where these tau species are easily measurable with current technologies and provide good diagnostic performance [29]. Early attempts to measure t-tau in blood have rendered mixed results, showing large overlaps between groups [36–39] and weak correlations between plasma and CSF concentrations [14, 36, 38]. A recently developed assay referred to as N-terminal fragment of tau or NT1 has shown high performance in plasma by identifying AD and predicting cognitive decline and neurodegeneration [17, 40, 41]. Additionally, CSF measurements of non-phosphorylated tau fragments belonging to the microtubule-binding region (MTBR) such as Tau368 or MTBR-tau243 were demonstrated to be associated with tau deposition in AD [11, 42, 43]. Interestingly, several blood phosphorylated and non-phosphorylated tau assays shared a similar design: an N-terminally directed strategy; that is targeting tau fragments that extend from the N-terminus to the mid-region. Illustrative examples of N-terminal directed p-tau assays include ADx p-tau181, Janssen p-tau217 and UGOT p-tau231, all of which show similar performance in CSF and blood [16]. In terms of N-terminally directed t-tau assays, Chen et al. showed that plasma NT1 (Tau12 [6-18aa] and BT2 [194-198aa]) performed better than NT2 (Tau12 [6-18aa] and Adx202 [218-224aa]), a longer t-tau assay expanding further into the mid-region [17]. Thus, given the promising results obtained by targeting N-terminal bearing tau fragments (both phosphorylated and non-phosphorylated), NTA (Tau12 [6-18aa] and HT7 [159-163aa]) was designed as an assay that could be used to exploit the potentially superior performance of targeting N-terminal containing tau fragments.

In a recent publication, we reported the validation and characterization of three novel in-house developed Simoa immunoassays capable of quantifying different lengths of N-terminal tau fragments in CSF. Among them, only NTA, an assay targeting N-terminal containing tau fragments (NTA-tau), was able to successfully identify AD in a small pilot plasma cohort [18]. This was followed by another study, where we investigated NTA-tau in a small albeit well-characterized cohort comprising by both CSF and plasma samples. Here, we demonstrated that NTA-tau is more tightly associated with tau-PET in AD than Aβ-PET and MRI neurodegeneration measurements, and that NTA-tau can track tau deposition in cognitively impaired amyloid-β positive individuals [44]. Therefore, in the present study, our aim was to expand previous findings, and to further characterize plasma NTA-tau by investigating how AD-related cerebral pathological changes (Aβ pathology, tau pathology, and neurodegeneration) may drive the increase of these NTA-tau in blood. We also assessed the association of plasma NTA-tau concentrations with two measures of cognitive performance. Moreover, we evaluated whether plasma NTA-tau concentrations can predict longitudinal changes in tau-PET, cortical thickness, and cognition. Finally, we also investigated the associations between longitudinal changes in plasma NTA-tau and baseline Aβ status, as well as longitudinal cortical thickness and longitudinal cognition. For this purpose, we investigated plasma NTA-tau in the Swedish BioFINDER-1 and BioFINDER-2 studies, both well-characterized by clinically validated fluid and imaging biomarkers, including cross-sectional and longitudinal samples of participants across the AD *continuum*, non-AD cases and control individuals.

## Methods

### Participants

Participants from two different cohorts were included in this study:, the BioFINDER-2 (NCT03174938) and BioFINDER-1 (NCT01208675), (Lund University, Lund, Sweden). Participants from both cohorts were recruited at the Skåne University Hospital and the Hospital of Ängelholm in Sweden. Further details on recruitment, inclusion and exclusion criterion are described elsewhere [35, 45]. All participants underwent a lumbar puncture at baseline, from which we obtained CSF Aβ42/40 and were classified as Aβ positive or negative (see below). Participants were then divided in either Aβ negative or positive cognitively unimpaired (CU- and CU + , respectively), Aβ positive mild cognitive impairment (MCI +), Aβ positive AD dementia (AD +), cognitively impaired nonAD patients, either Aβ positive (nonAD +) or negative (nonAD-). Diagnosis was determined by consensus of memory clinic physicians for all participants. MCI diagnosis was established if participants performed below 1.5 standard deviation from age and education stratified norms on at least one domain from an extensive neuropsychological battery examining memory, verbal, visuospatial, and attention/executive domains [35]. For AD dementia, diagnosis was based on the criteria from the Diagnostic and Statistical Manual of Mental Disorders Fifth Edition and if positive on Aβ biomarkers based on the updated NIA-AA criteria for AD [5]. Dementia cases were only available in BioFINDER-2. Participants diagnosed as non-AD cognitive impairment fulfilled the criteria for dementia or minor neurocognitive disorder due to frontotemporal dementia, Parkinson's disease, vascular dementia, dementia with Lewy bodies, progressive supranuclear palsy, multiple system atrophy, corticobasal syndrome, or primary progressive aphasia.

All participants included in this study had at least one plasma NTA-tau measurement available. In all BioFINDER-2 participants, plasma p-tau181, glial fibrillary acidic protein (GFAP) and neurofilament light (NfL) were available. In a subset of these participants (*n* = 715), plasma total tau was also available. Some of these BioFINDER-2 participants had also available longitudinal measures of imaging and cognition. Some BioFINDER-1 participants had longitudinal plasma NTA-tau measures and some longitudinal measures of cortical thickness (*n* = 681, mean(SD) time = 6.9(2.2) years) and cognition (*n* = 442, mean(SD) time = 4.9(1.8) years).

All participants gave written informed consent and ethical approval was granted by the Regional Ethical Committee in Lund, Sweden.

### Plasma and CSF biomarker measurements

For most BioFINDER-2 participants (*n* = 1,294) and all BioFINDER-1 (*n* = 932), CSF levels of Aβ42 were measured using the Elecsys β-Amyloid (1–42), electrochemiluminescence immunoassays on a fully automated cobas e 601 instrument (Roche Diagnostics International Ltd., Rotkreuz, Switzerland) and CSF Aβ40 levels were measured with robust prototype assays as part of the Roche NeuroToolKit on cobas e 601 instruments (Roche Diagnostics International Ltd, Rotkreuz, Switzerland). For the rest of BioFINDER-2 participants, the CSF Aβ42/40 ratio was obtained through clinical measurements (Lumipulse or Meso Scale Discovery) for assessing Aβ positivity (for cut-offs, see below). Only measurements with Roche instruments were used for analyses with continuous CSF Aβ42/40 levels.

Plasma NTA-tau levels in BioFINDER-2 and in BioFINDER-1 cohorts were quantified using an in-house developed immunoassay using a Simoa HD-X platform (Quanterix) at the Clinical Neurochemistry Laboratory, Sahlgrenska University Hospital, Mölndal (Sweden). NTA assay development and validation have been previously described elsewhere [18]. The name “NTA-tau” emphasizes the immunoassay design, intended to target N-terminal containing tau species, but it is important to note that this does not exclude the possibility of the assay binding long tau species containing other tau regions, as long as they include the N-terminus. Briefly, NTA Simoa assay is comprised by a mouse monoclonal antibody against mid-region tau and used as capture antibody. Biotinylated mouse monoclonal antibody against N-terminal tau was used for detection. Randomized plasma samples were allowed to thaw for 45 min at room temperature, after which they were vortexed and centrifuged at 4000 g for 10 min. Samples were then diluted 1:2 using commercially available Tau2.0 assay diluent (Quanterix). Eight-point calibration curves were generated using commercially available non-phosphorylated recombinant full-length tau411 (SignalChem) and run in duplicates. Internal quality controls samples were included in all plates before and after the samples and run in duplicates. Repeatability and intermediate precision values in the cohort was < 15%.

Other plasma biomarkers were used for comparison in some analyses in BioFINDER-2. Plasma p-tau181 was measured at Lund University using an immunoassay on the Meso Scale Discovery platform developed by Lilly Research Laboratories [46]. Plasma NfL, GFAP and t-tau were quantified at the Clinical Neurochemistry Laboratory, Sahlgrenska University Hospital, Mölndal (Sweden) using Simoa (Quanterix) assays. These measurements have been previously published and used here for comparison purposes [47].

### Imaging measures

Description of imaging acquisition and processing has been detailed before for BioFINDER-2 and BioFINDER-1 [35, 48]. In BioFINDER-2, Aβ- and tau-PET were acquired after 90–110 min after the injection of ~ 185 MBq [^18^F]flutemetamol and after 70–90 min post injection of ~ 370 MBq [^18^F]RO948, respectively. Of note, most AD dementia patients did not undergo Aβ-PET imaging due to study design. In BioFINDER-1, Aβ-PET was also acquired using[^18^F]flutemetamol but no tau-PET was available. To assess neurodegeneration, we used cortical thickness from structural magnetic resonance image (MRI) acquired with high resolution T1-weighted anatomical magnetization-prepared rapid gradient echo (MPRAGE) images (1mm isotropic voxels) in both studies [49]. T1-images underwent volumetric segmentation and parcellation using FreeSurfer (v.6.0, https://surfer.nmr.mgh.harvard.edu), which were also used for PET quantification after the registration and normalisation processes. For main analyses, we measured the variables of interest in specific regions known to be specifically affected in AD. For Aβ-PET, we calculated mean Standardized uptake value ratio (SUVR) in a neocortical meta-region of interest (ROI) similar to the Centiloid mask using the whole cerebellum as reference region [50]. For tau-PET, mean SUVR was calculated in a temporal meta-ROI (Braak I-IV) [51] with the inferior cerebellum as reference region. The AD-specific cortical thickness meta-ROI encompassed temporal regions with known susceptibility to atrophy in AD as previously described [52]. For additional analyses, we also calculated these values in all FreeSurfer regions averaging the two hemispheres to reduce the number of comparisons. For subcortical regions we used volumes, instead of thickness, with the neurodegeneration-related analyses.

### Cognitive measures

In both cohorts, Mini-mental state examination (MMSE) was used as a measure of global cognition as it is widely used in the clinical setting. Further, we also derived a modified version of the preclinical Alzheimer’s cognitive composite (mPACC), as a more sensitive measure of cognitive decline, especially in early stages, typically used in the research setting. The mPACC was calculated as the average of four z-scores. For tests of memory, the 10-word delayed recall task from the Alzheimer’s Disease Assessment Scale-Cognitive subscale [ADAS-cog]) was used, weighted twice, to preserve the weight of memory tests in the original PACC [53], for verbal ability animal fluency was used, for executive function Trail Making Test A [TMT-A], and for global cognition, the MMSE was used, as previously described [54].

### Cohort stratification: AT groups and braak stages

Besides clinical diagnosis, participants were stratified based on the presence of Aβ (A, determined using CSF Aβ42/40) and tau pathology (T, determined using tau-PET) into A-T-, A+T-, A+T+ and the A-T+ groups. For CSF Aβ42/40 we used previously validated cut-offs specifics for each platform (Elecsys: 0.08, Innotest: 0.752, Lumipulse: 0.72, MSD: 0.752). Tau-PET was categorized based on the SUVR in the meta-temporal ROI (Braak I-IV: 1.32) [55]. Participants with available tau-PET imaging were stratified according to PET Braak stages into Braak 0, Braak I-II, Braak III-IV, and Braak V-VI in a hierarchical manner, based on regional cut-offs (Braak I-II:1.38, Braak III-IV: 1.32, Braak V-VI: 1.19). As additional analysis, we also classified participants based on the recent AA diagnostic criteria (https://aaic.alz.org/diagnostic-criteria.asp). We used the region Braak I-II for the medial temporal lobe (MTL) classification using the previously mentioned cutoff. For the neocortical region we used the multiblock barycentric discriminant analysis (MUBADA) region, using previously validated cutoffs (intermediate: 1.10 < SUVR ≤ 1.46, high: SUVR > 1.46) [56]. Aβ status was based on Aβ PET when available, or CSF Aβ42/40 ratio, due to the lack of Aβ PET on dementia cases.

### Statistical analysis

We performed different set of analyses in the two cohorts according to the available data in each case. ANCOVA was used to assess differences by diagnosis (clinical framework, both cohorts), Aβ and tau status (A/T, BioFINDER-2) and Braak stages (research framework, BioFINDER-2), adjusting for age and sex. *Post-hoc* comparisons were performed with the Tukey’s multiple comparison test. In the Braak and AA diagnostic criteria classification analyses, participants not following the hierarchical model were excluded. Box plots include all participants, displaying the median and the interquartile range; whiskers show the lower value of maximum/minimum value or 1.5 interquartile range from the hinge. Additionally, in BioFINDER-2, we also checked whether chronic kidney disease (CKD) influenced plasma NTA-tau levels using linear regression models adjusting for age, sex and diagnosis. Linear regression models were used to assess the association between Aβ, tau or neurodegeneration (outcome) and plasma levels (predictor) in independent models with age and sex as covariates. We compared models including/excluding an interaction between plasma NTA-tau and Aβ-status using R^2^ and the corrected Akaike information criteria (AICc) and report the optimal ones. All participants with available Aβ-PET (both cohorts) or tau-PET (BioFINDER-2) were included in such analyses, but we excluded non-AD patients when looking at neurodegeneration (both cohorts) to avoid bias. We also assessed the association between plasma NTA-tau and cognition in Aβ-positive participants (both cohorts), excluding non-AD patients, using linear regression models adjusting for age, sex and years of education. Receiver operating curves (ROC) were used to assess the usefulness of plasma NTA for categorising participants for Aβ and tau status (*pROC* package). We report area under the curve (AUC), and sensitivity and specificity at the optimal outpoint based on Youden’s index. Multivariable linear regression models were used to assess the optimal model for explaining plasma NTA-tau levels (ln-transformed). Aβ (CSF Aβ42/40), tau (PET, ln-transformed) and neurodegeneration (cortical thickness) were used as predictors in different models with age and sex as covariates (BioFINDER-2). Three models with a unique predictor were constructed, then we created two further models with two predictors that are supposed to happen consecutively (*i.e.*, Aβ and tau or tau and neurodegeneration), and a final model with all three predictors. We also constructed an additional model with only covariates. All these seven models were compared based on the AICc (*MuMIn* package) to select the one that best explained plasma NTA-tau levels, avoiding over-fitting. The optimal model was selected as that with the minimal AICc. Comparison to simpler models was performed with an F-test. From the optimal model, we then calculated the proportion of variation explained by each predictor using partial R^2^ with the *sensemakr* package. Differences between Aβ and tau partial R^2^ were assessed by bootstrapping. We excluded nonAD participants from this analysis to avoid bias in the neurodegeneration marker due to other neurodegeneration diseases. This analysis was repeated in the other sets of plasma biomarkers (first set [*n* = 1,294]: NTA-tau, p-tau181, GFAP and NfL, second set [*n* = 715]: NTA-tau and t-tau) for comparison.

For longitudinal analyses, we first used linear mixed models (*lme4* package) for assessing the association between baseline plasma NTA-tau levels and tau accumulation, brain atrophy or cognitive decline. Tau-PET binding (BioFINDER-2), cortical thickness (both cohorts) or cognition (MMSE or mPACC, both cohorts) were used as outcomes in independent models with interaction between plasma levels and time was used as predictor and age and sex (and education years for cognition) as covariates. Random intercepts and random time slopes were included in the models. Only participants within the AD *continuum* were included in these analyses as they are those supposed to progress. For BioFINDER-1 participants, we also had available longitudinal plasma NTA-tau measures, which were used to assess how they were related to disease stage and progression First, we evaluated how these plasma NTA-tau levels changed over time by Aβ-status at baseline (*i.e.*, positive/negative) at baseline using linear mixed models. Plasma NTA-tau levels were used as outcomes and interaction between Aβ-status and time was used as predictor, with random slopes and intercepts, adjusting for age and sex. Finally, we also evaluated whether changes in plasma NTA-tau levels were associated with changes in disease progression (*i.e.*, atrophy and cognitive decline). We first derived plasma NTA-tau slopes using linear mixed models with random slopes and intercepts including only time as predictor. Then we used the interaction between time and these slopes as predictors, in another linear mixed model with cortical thickness or cognition as outcome. In these last models age and sex (and education in the case of cognition) were also included as covariates.

Plasma NTA-tau levels, as well as Aβ- and tau-PET measures were log-10 transformed in all correlation analyses. R Studio (v.4.1.0) was used both statistical analysis and visualizations. For main analyses, statistical significance was set at *p* < 0.05 uncorrected for multiple comparisons unless stated. For regional analyses, statistical significance was set at *p* < 0.05 corrected for multiple comparisons using false-discovery rate (FDR).

## Results

### Demographics

For BioFINDER-2, a total of 1,294 participants had available plasma NTA-tau measures (Table 1). From these, 628 were CU, out of which 466 were Aβ-negative (CU-) and 162 were Aβ-positive (CU+); 148 were classified as having MCI and 189 having dementia due to AD, all Aβ-positive; and 329 were classified as having a non-AD cognitive impairment, out of which 79 were Aβ-positive (nonAD+) and 250 were Aβ-negative (nonAD-). The mean (SD) age of the sample was 67.8 (12.5) years, there were a total of 623 (48.1%) women and 614 (47.4%) *APOE* ε4 carriers. All these participants had plasma NTA-tau levels available as well as plasma p-tau181, plasma GFAP and plasma NfL. A subset of 715 participants (Supplementary Table 1) also had available plasma t-tau. In BioFINDER-2, the effect of chronic kidney disease was examined and found to be significant (β[95%CI] = 0.54[0.39, 0.70], *p* < 0.001) on plasma NTA-tau when adjusting for age, sex and diagnosis. For BioFINDER-1, a total of 932 participants had available plasma NTA-tau measures (Table 1). Out of these, 495 were CU-, 192 were CU+ , 155 were classified as MCI+ and 90 were MCI Aβ-negative (MCI-). Here, the mean age was 72.0 (5.4) years, there were 532 (57.1%) women and 273 (29.3%) *APOE* ε4 carriers.

**Table 1:** Characteristics of the sample

	**BIOFINDER-2**								**BIOFINDER-1**					
	All (*n* = 1,294)	CU- (*n* = 466)	CU+ (*n* = 162)	MCI+ (*n* = 148)	AD+ (*n* = 189)	nonAD + (*n* = 79)	nonAD- (*n* = 250)	*p*	All (*n* = 932)	CU- (*n* = 495)	CU+ (*n* = 192)	MCI+ (*n* = 155)	MCI- (*n* = 90)	*p*
**Age**	67.8 (12.5)	60.3 (15.2)	71.6 (9.2)***	72.6 (6.8)***	73.4 (6.9)***	74.6 (6.1)***	70.3 (9.1)***	< 0.001	72.0 (5.4)	71.8 (5.4)	73.2 (5.4)**	72.7 (5.1)*	69.9 (5.6)*	< 0.001
**Women, n(%)**	623 (48.1%)	251 (53.9%)	82 (50.6%)	66 (44.6%)***	107 (56.6%)	27 (34.2%)***	90 (36.0%)***	< 0.001	532 (57.1%)	313 (63.2%)	119 (62.0%)	73 (47.1%)**	27 (30.0%)***	< 0.001
***APOE*** ε***-4* carriers, n(%)**^**a,g**^	614 (47.4%)	167 (35.8%)	100 (61.7%)***	106 (71.6%)***	134 (70.9%)***	49 (62.0%)***	58 (23.2%)***	< 0.001	273 (29.3%)	55 (11.1%)	91 (47.4%)***	108 (69.7%)***	19 (21.1%)	< 0.001
**Education, years**^**b,h**^	12.7 (3.8)	13.0 (3.4)	12.6 (3.7)	12.9 (4.7)	12.2 (4.1)**	13.0 (4.2)	12.0 (3.7)***	< 0.001	11.9 (3.5)	12.2 (3.4)	12.1 (3.8)	11.2 (3.4)**	11.0 (3.4)**	< 0.001
**Imaging measures**														
**Centiloids**^**c,i**^	16.9 (41.0)	-7.50 (7.4)	46.0 (37.8)***	74.3 (39.6)***	95.6 (29.0)***	42.3 (45.6)	-5.00 (10.2)*	< 0.001	37.0 (45.1)	2.06 (7.6)	58.4 (38.5)***	85.9 (34.6)***	5.51 (16.5)	< 0.001
**Tau-PET SUVR**^**d**^	1.34 (0.44)	1.13 (0.09)	1.28 (0.29)***	1.50 (0.44)***	2.11 (0.61)***	1.28 (0.20)***	1.16 (0.09)***	< 0.001	-	-	-	-	-	-
**Cortical thickness**^**J**^	2.51 (0.15)	2.60 (0.11)	2.54 (0.12)***	2.47 (0.11)***	2.35 (0.14)***	2.37 (0.16)***	2.52 (0.14)***	< 0.001	2.52 (0.20)	2.58 (0.16)	2.53 (0.20)	2.41 (0.22)***	2.49 (0.22)**	< 0.001
**Cognition**														
**MMSE**^**e**^	26.7 (4.00)	29.0 (1.16)	28.7 (1.34)**	26.7 (1.86)***	20.8 (4.31)***	23.5 (5.51)***	26.4 (3.35)***	< 0.001	28.4 (1.7)	29.0 (1.1)	28.7 (1.4)*	26.6 (1.8)***	27.5 (1.9)***	< 0.001
**mPACC**^**f**^	-1.19 (1.88)	0.18 (0.69)	-0.28 (0.76)***	-1.96 (0.90)***	-3.99 (1.75)***	-2.70 (2.04)***	-1.74 (1.57)***	< 0.001	-0.80 (1.16)	-0.15 (0.74)	-0.49 (0.83)***	-2.14 (1.00)***	-1.54 (0.90)***	< 0.001
**Plasma levels**														
**Plasma NTA**	0.258 (0.165)	0.205 (0.112)	0.266 (0.150)***	0.285 (0.145)***	0.444 (0.212)***	0.239 (0.133)*	0.204 (0.128)	< 0.001	0.132 (0.123)	0.114 (0.113)	0.140 (0.0941)***	0.189 (0.154)***	0.118 (0.134)	< 0.001

*P*-values show differences among groups as calculated with Kruskal–Wallis or Chi-squared tests. *Post-hoc* analyses against CU- group are shown in the cells. *: *p* < 0.05; **: *p* < 0.01; ***: *p* < 0.001. Mean (SD) is reported unless otherwise indicated

*Abbreviations*: *AD+* Alzheimer’s dementia Aβ-positive, *A-T-* Aβ and tau negative, *A+T-* Aβ-positive tau negative, *A+T+* Aβ and tau positive, *A-T+* Aβ-negative tau positive, *CU-* cognitively unimpaired Aβ-negative, *CU+* cognitively unimpaired Aβ-positive, *MCI+* mild cognitive impairment Aβ-positive, *MMSE* Mini-Mental State Examination, *nonAD+* non-Alzheimer’s type dementia Aβ-positive, *nonAD-* non-Alzheimer’s type dementia Aβ-negative, *mPACC* modified preclinical Alzheimer’s cognitive composite, *SUVR* standardized uptake value ratio

^a^ 84 participants missing in BioFINDER-1

^b^ 29 participants missing in BioFINDER-1

^c^ 454 participants missing in BioFINDER-1

^d^ 113 participants missing in BioFINDER-1

^e^ 5 participants missing in BioFINDER-1

^f^ 77 participants missing in BioFINDER-1

^g^ 221 participants missing in BioFINDER-2

^h^ 7 participants missing in BioFINDER-2

^i^ 718 participants missing

^j^ 261 participants missing

^k^ 243 participants missing

### Plasma NTA-tau concentrations across clinical diagnosis and disease stages

**Fig. 1:**
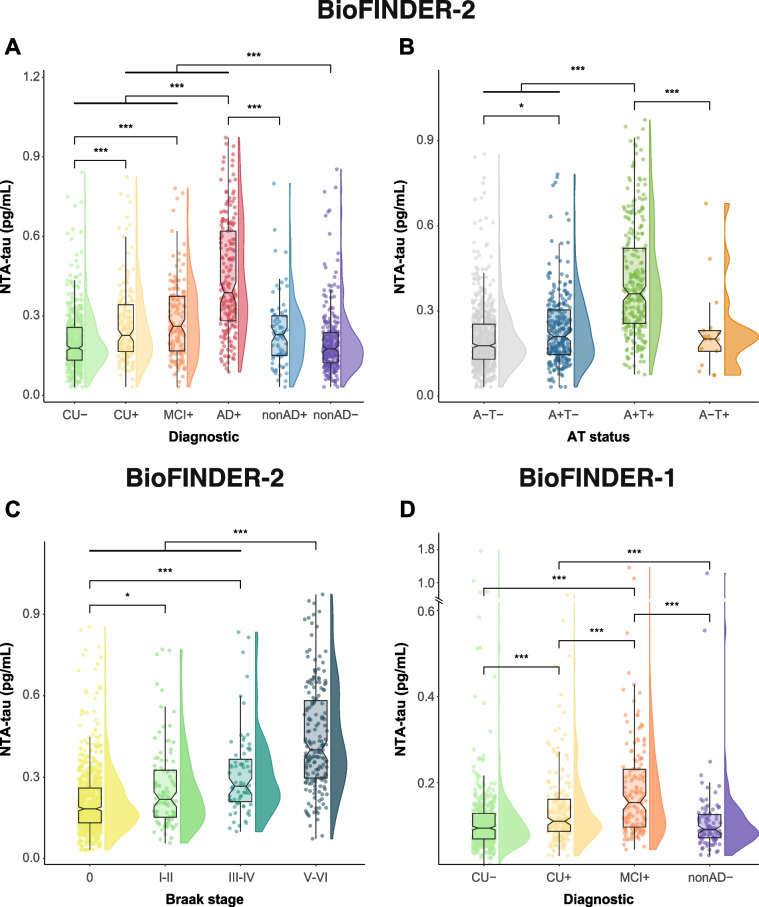
Plasma NTA-tau levels across clinical diagnosis and disease stages. Plasma NTA-tau levels in BioFINDER-2 by clinical diagnosis (**A**), A/T status (**B**) and Braak stages (**C**). Plasma NTA-tau levels in BioFINDER-1 by clinical diagnosis (**D**). Differences of plasma levels by diagnostic groups were measured using ANCOVA and Tukey’s method for *post-hoc* comparisons. Age and sex were used as covariates in all cases. Aβ (A) status was assessed using CSF Aβ42/40 levels and tau (T) status using tau-PET SUVR based on previously validated cut-offs. Participants with available tau-PET imaging were stratified according to the PET Braak stages in a hierarchical manner, based on regional SUVR cut-offs. In D we divided the y-axis to show few cases with very high plasma NTA-tau levels. *: *p* < 0.05; **: *p* < 0.01; ***: *p* < 0.001. Box plots include all participants, displaying the median and the interquartile range; whiskers show the lower value of maximum/minimum value or 1.5 interquartile range from the hinge. Abbreviations: Aβ, amyloid-β; AD+ , Alzheimer’s dementia Aβ-positive; A-T-, Aβ and tau negative; A+T-, Aβ-positive tau negative; A+T+ , Aβ and tau positive; A-T+ , Aβ-negative tau positive; CSF, cerebrospinal fluid; CU-, cognitively unimpaired Aβ-negative; CU+ , cognitively unimpaired Aβ-positive; MCI+ , mild cognitive impairment Aβ-positive nonAD+ ; non-Alzheimer’s type dementia Aβ-positive; non-AD-, non-Alzheimer’s type dementia Aβ-negative; SUVR, standardized uptake value ratio

We first investigated plasma NTA-tau concentrations across clinical diagnosis groups in BioFINDER-2 (Fig. 1A and Supplementary Table 2). Increased levels of plasma NTA-tau were exclusively seen in Aβ-positive groups, where it progressively increased across the AD *continuum* (CU+ , MCI+ and AD+), especially in AD+ *.* Plasma NTA-tau starts increasing in preclinical AD cases, being significantly increased in CU+ compared with CU- (*p* = 0.001). Plasma NTA-tau was also increased in MCI+ when compared with CU- individuals (*p* < 0.001). No significant differences were observed between CU+ and MCI+ , albeit plasma NTA-tau levels seemed slightly higher in the latter group. Plasma NTA-tau was pronouncedly increased in AD+ cases showing significantly higher levels than all Aβ-negative and Aβ positive groups (*p* < 0.001 for all). Plasma NTA-tau was significantly increased in CU+ and MCI+ compared with nonAD- (*p* < 0.001 for all). No significant differences in plasma NTA-tau levels were observed between nonAD+ , nonAD- and CU- cases.

BioFINDER-2 participants were also stratified into AT groups according to the presence/absence Aβ pathology (A, determined by CSF Aβ42/40) and tau pathology (T, determined by tau-PET, Supplementary Table 3). Plasma NTA-tau increased progressively across the AD *continuum*: A modest yet significant increase in plasma NTA-tau levels was observed between A-T- and A+T- (*p* = 0.022, Fig. 1B and Supplementary Table 4). This was followed by a pronounced increase between A+T- and A+T+ (*p* < 0.001). Plasma NTA-tau was also significantly higher in A+T+ compared with A-T- (*p* < 0.001).

Additionally, we examined the levels of plasma NTA-tau across BioFINDER-2 participants stratified by Braak stages (Supplementary Table 5). Plasma NTA-tau showed a moderate increase from Braak 0 to I-II (*p* = 0.016, Fig. 1C and Supplementary Table 6). Plasma NTA-tau levels were significantly higher in Braak III-IV compared with Braak 0 (*p* < 0.001) but not when compared with Braak I-II (although they approached statistical significance, *p* = 0.088). The most prominent increase occurred between Braak III-IV and V-VI, with Braak V-VI subjects displaying the highest plasma NTA-tau levels and being increased compared with all groups (*p* < 0.001 for all). As a supplementary analysis, plasma NTA-tau levels were also evaluated by classifying participants according to the recently proposed Alzheimer's Association criteria for staging AD using PET. Plasma NTA-tau was increased in PET stages positive for both Aβ and tau-PET, that is A+MTL+ N-, A+MTL+N+ and A+MTL+N+ + (*p* < 0.001, for all), with the latter group clearly displaying the highest NTA-tau concentrations (Supplementary Fig. 1 and Supplementary Table 7).

In BioFINDER-1, tau-PET was not available so we could only test differences across clinical diagnosis groups (Fig. 1D and Table 1). As observed in BioFINDER-2, NTA-tau increased progressively across Aβ-positive groups, being significantly increased in MCI+ compared with CU+ (*p* < 0.001). Plasma NTA-tau levels were also significantly higher in CU+ and MCI+ compared with both CU- and nonAD- cases (*p* < 0.001, for all, Supplementary Table 8).

### Cross-sectional associations between plasma NTA-tau and Aβ-PET, tau-PET and cortical thickness

**Fig. 2:**
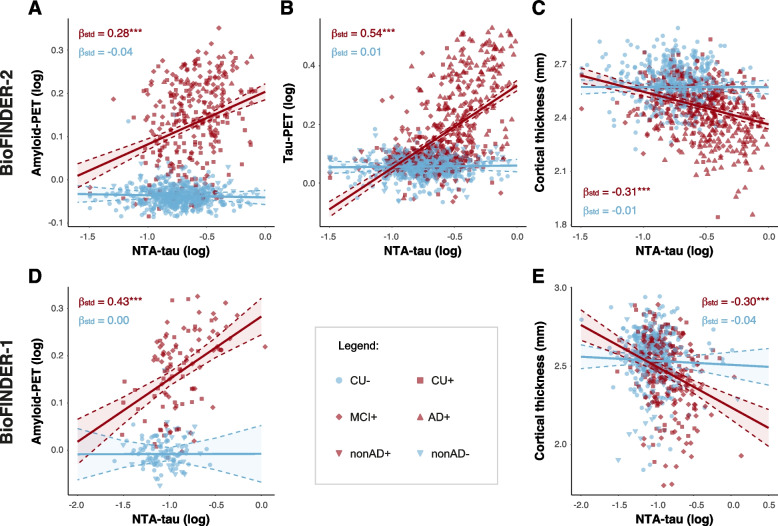
Cross-sectional associations between plasma NTA-tau and Aβ-PET, tau-PET and cortical thickness. Cross-sectional associations between plasma NTA-tau levels and Aβ-PET (**A**, **D**), tau-PET (**B**) and cortical thickness (**C**, **E**) by Aβ-status in BioFINDER-2 (**A**, **B** and **C**) and BioFINDER-1 (D and E). Linear regressions with plasma NTA-tau levels as predictor were used to measure the association with Aβ-PET (Centiloids), tau-PET (SUVR), cortical thickness (AD-signature). Standardized β (β_std_) and p-values of the associations for each group are shown in the plot coloured accordingly (red: Aβ-positive, blue: Aβ-negative). Age and sex were used as covariates in all cases. Non-AD patients were excluded in the analyses with cortical thickness. Aβ-status was determined using CSF Aβ42/40. *: *p* < 0.05; **: *p* < 0.01; ***: *p* < 0.001. Abbreviations: Aβ, amyloid-β; AD, Alzheimer’s disease; FDR, false discovery rate; nonAD+ ; non-Alzheimer’s type dementia Aβ-positive; SUVR, standardized uptake value ratio

Next, we tested associations between plasma NTA-tau levels and imaging markers of insoluble Aβ and tau aggregates, using Aβ- and tau-PET (only available in BioFINDER-2), respectively, and neurodegeneration, using MRI measurements of cortical thickness. Models including an interaction between plasma NTA-tau levels and Aβ-status were selected as being statistically better (Supplementary Table 9). Looking at global measures in BioFINDER-2, we observed a significant different association (*p* < 0.001 all cases) between Aβ-positive and Aβ-negative participants in all three cases. In particular, only Aβ-positive participants showed a significant association between plasma NTA-tau and Aβ-PET (β[95%CI] = 0.28[0.18, 0.39], *p* < 0.001), tau-PET (β[95%CI] = 0.54[0.46, 0.61], *p* < 0.001) and cortical thickness (β[95%CI] = -0.31[-0.40, -0.23], *p* < 0.001) (Fig. 2A-C and Table 2). Similar findings were observed in BioFINDER-1 participants for Aβ-PET (β[95%CI] = 0.43[0.25, 0.61], *p* < 0.001) and cortical thickness (β[95%CI] = -0.30[-0.41, -0.20], *p* < 0.001) (Fig. 2D-E and Table 2).

**Table 2:** Associations between plasma NTA-tau and Aβ-PET, tau-PET, cortical thickness, and cognition cross-sectionally in BioFINDER-2 and BioFINDER-1

NTA associations with:		β [95%CI] Aβ-negative	p Aβ-negative	β [95%CI] Aβ-positive	p Aβ-positive
BIOFINDER-2	Aβ-PET	-0.04 [-0.12, 0.04]	0.372	0.28 [0.18, 0.39]	< 0.001
	Tau-PET	0.01 [-0.06, 0.08]	0.772	0.54 [0.46, 0.61]	< 0.001
	Cortical thickness	-0.01 [-0.09, 0.08]	0.845	-0.31 [-0.40, -0.23]	< 0.001
	MMSE	-	-	-0.46 [-0.54, -0.38]	< 0.001
	mPACC	-	-	-0.38 [-0.46, -0.30]	< 0.001
BIOFINDER-1	Aβ-PET	0 [-0.19, 0.19]	0.989	0.43 [0.25, 0.61]	< 0.001
	Cortical thickness	-0.04 [-0.14, 0.05]	0.388	-0.30 [-0.41, -0.20]	< 0.001
	MMSE	-	-	-0.52 [-0.71, -0.33]	< 0.001
	MPACC	-	-	-0.41 [-0.54, -0.28]	< 0.001

Linear regressions with plasma NTA-tau levels as predictor were used to measure the association with Aβ-PET (SUVR), tau-PET (SUVR), cortical thickness (AD-signature) and cognition (MMSE and mPACC) by Aβ-status. Age and sex (and education for cognition) were used as covariates. Non-AD patients were excluded in the analyses with cortical thickness and cognitive measures

*Abbreviations*: *Aβ* amyloid-β, *AD* Alzheimer’s disease, *MMSE* Mini-Mental State Examination, *nonAD+* non-Alzheimer’s type dementia Aβ positive, *mPACC* modified preclinical Alzheimer’s cognitive composite, *SUVR* standardized uptake value ratio

In BioFINDER-2 participants, where all three imaging modalities were available, we repeated these same analyses regionally and we compared it with the other plasma biomarkers (Supplementary Fig. 2). Plasma NTA-tau was the biomarker showing the strongest regional association with tau-PET and cortical thickness, and these being especially prominent in temporo-parietal areas for both tau deposition and brain atrophy. Finally, plasma NTA-tau regional association with Aβ-PET was weaker than those of plasma p-tau181 and GFAP, but stronger than that of plasma NfL. Additionally, we investigated the diagnostic accuracy of plasma NTA-tau when discriminating Aβ-PET and tau-PET status. Plasma NTA-tau performance discriminating Aβ-PET was AUC_NTA_[95%CI] = 0.67 [0.63–0.71] (Sensitivity = 0.46, Specificity = 0.79). When discriminating tau-PET, plasma NTA-tau had and AUC of AUC_NTA_[95%CI] = 0.80 [0.77–0.83] (Sensitivity = 0.68, Specificity = 0.78).

### Proportion of variation in plasma NTA-tau levels explained by Aβ, tau and neurodegeneration

**Fig. 3:**
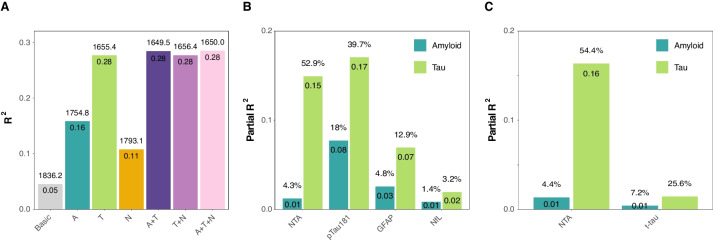
Proportion of variation in plasma NTA levels explained by Aβ, tau and neurodegeneration. Performance of different models for predicting plasma NTA-tau levels are shown in **A**. Each barplot represents one independent model, including CSF Aβ42/40 (A), tau-PET SUVR (T) and/or cortical thickness (N) as predictors in multivariable models. All models are adjusted for age and sex. Basic model includes only covariates. R^2^ values are shown inside the barplots and AICc of each model is shown on top. The optimal model predicting plasma NTA-tau was A&T because it had the highest R^2^ and the lowest AICc. Partial R^2^ of Aβ (CSF A β42/40) and tau (tau-PET) for predicting NTA-tau levels are shown in **B** (all participants, *n* = 1,294) and **C** (subsample with t-tau, *n* = 715). Light green bars represent the variance explained by tau and dark green bars represent that explained by Aβ. Other plasma biomarkers available are shown for comparison. Partial R^2^ values are shown inside the bars and percentage of the total R^2^ of the model are shown on top. In all cases, age and sex were used as covariates. NonAD participants were excluded from these analyses to avoid bias from neurodegeneration markers. Abbreviations: Aβ, amyloid-β; AICc, corrected Akaike criterion; CSF, cerebrospinal fluid; nonAD, non-Alzheimer’s type dementia; SUVR, standardized uptake value ratio

We next investigated the proportion of variation in plasma NTA-tau levels explained by Aβ pathology (A, CSF Aβ42/40), tau pathology (T, tau-PET) and neurodegeneration (N, MRI cortical thickness) in BioFINDER-2 participants. We observed that a model with both Aβ and tau pathologies (A&T) optimally explained plasma NTA-tau levels (R^2^ = 0.28, AICc = 1649.5). Although the simpler tau-only model explained variance to a similar extent (R^2^ = 0.28, AICc = 1655.4, Fig. 3A), the model including both A&T was significantly better (F = 7.96, *p* = 0.005).

Once the optimal model was selected (A&T), we investigated which proportion of variation was explained by each measure of pathology using partial R^2^. For comparison, we also performed the same analysis with the other plasma biomarkers available in the whole cohort (*i.e.*, p-tau181, GFAP and NfL). We observed that the proportion of variation explained for plasma NTA-tau by tau was significantly higher (partial R^2^ = 0.15, 52.9% of the total R^2^), than by Aβ, which was minimal (partial R^2^ = 0.01, 4.3% of the total R^2^, difference: *p* < 0.001, Fig. 3B and Supplementary Table 10), supporting the results of the previous analysis. On the other hand, p-tau181 levels were explained by both Aβ (p-tau181: partial R^2^ = 0.08, 18.0% of the total R^2^) and tau (p-tau181: partial R^2^ = 0.17, 39.7% of the total R^2^), although the variance explained by tau was significantly higher (*p* = 0.020). GFAP levels were similarly explained by Aβ (partial R^2^ = 0.03, 4.8% of the total R^2^) and tau pathology (partial R^2^ = 0.07, 12.9% of the total R^2^, difference: *p* = 0.109). Finally, NfL was poorly explained by either Aβ (partial R^2^ = 0.01, 1.4% of the total R^2^) and tau (partial R^2^ = 0.02, 3.2% of the total R^2^, *p* = 0.494). The only t-tau assay available for comparison with plasma NTA-tau was Quanterix plasma t-tau, but this was only available in the subset of the original population, comprising 714 participants. Within this subset, plasma NTA-tau was again mostly explained by tau (partial R^2^ = 0.16, 54.4% of the total R^2^) and minimally by Aβ (partial R^2^ = 0.01, 4.4% of the total R^2^, difference: *p* < 0.001), whereas plasma t-tau was poorly explained by both Aβ (partial R^2^ = 0.00, 7.2% of the total R^2^) and tau (partial R^2^ = 0.01, 25.6% of the total R^2^, difference: *p* = 0.454; Fig. 3C and Supplementary Table 10).

### Cross-sectional associations between plasma NTA-tau and cognition

**Fig. 4:**
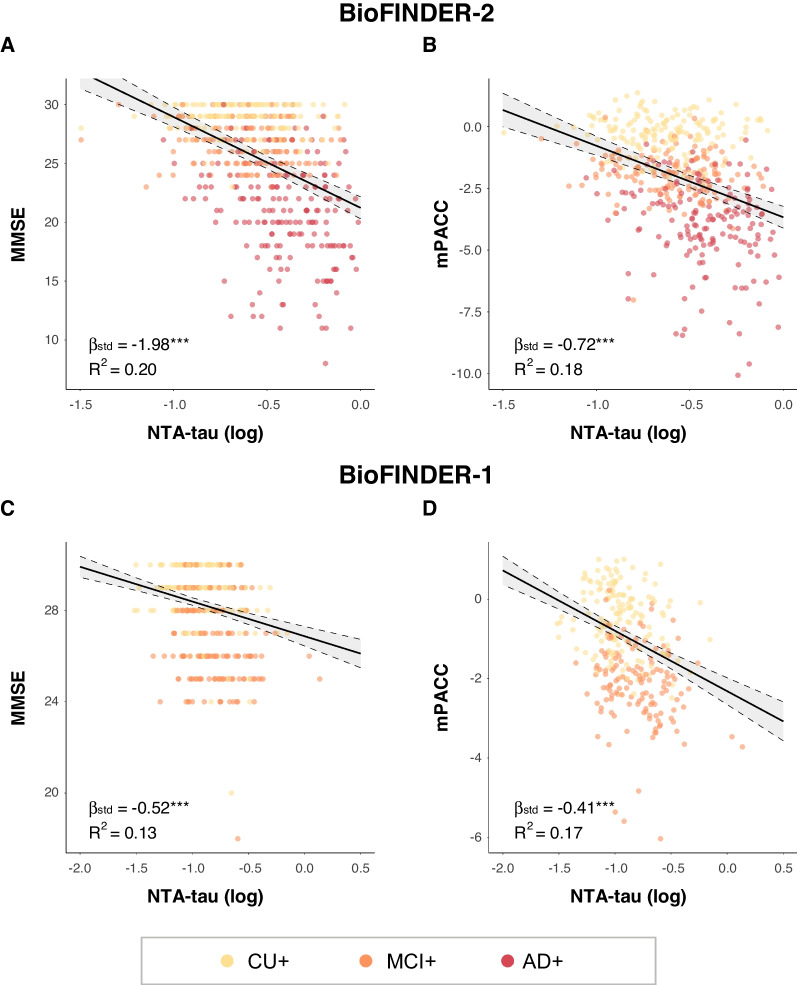
Cross-sectional associations between plasma NTA-tau and cognition. Cross-sectional associations between plasma NTA-tau levels and MMSE (**A** and **C**) and mPACC (**B** and **D**) in BioFINDER-2 (**A** and **B**) and BioFINDER-1 (**C** and **D**). Linear regressions with plasma NTA-tau levels as predictor and cognitive tests as outcome were used to measure the association. Age, sex and years of education were used as covariates in all cases. Only Aβ+ participants within the AD *continuum* (excluding nonAD+) were included in the analyses. Standardized β (β_std_) and p-values of the associations as well as the R^2^ of the model are shown in the plots. *: *p* < 0.05; **: *p* < 0.01; ***: *p* < 0.001. Abbreviations: Aβ, amyloid-β; AD, Alzheimer’s disease; MMSE, Mini-Mental State Examination; mPACC, mPACC, modified preclinical Alzheimer’s cognitive composite; nonAD+ ; non-Alzheimer’s type dementia Aβ-positive

Next, we cross-sectionally investigated the associations between plasma NTA-tau and cognition in both cohorts. In participants within the AD *continuum* (*i.e.*, CU+ , MCI+ and AD+), plasma NTA-tau was negatively associated with both MMSE (BioFINDER-2: β[95%CI] = -1.98[-2.34, -1.61], *p* < 0.001, R^2^ = 0.20; BioFINDER-1: β[95%CI] = -0.52[-0.71, -0.33], *p* < 0.001, R^2^ = 0.13) and mPACC (BioFINDER-2: β[95%CI] = -0.72[-0.89, -0.55], *p* < 0.001, R^2^ = 0.18; BioFINDER-1: β[95%CI] = -0.41[-0.54, -0.28], *p* < 0.001, R^2^ = 0.17) (Fig. 4A-D and Table 2).

### Associations between baseline plasma NTA-tau and disease progression

**Fig. 5:**
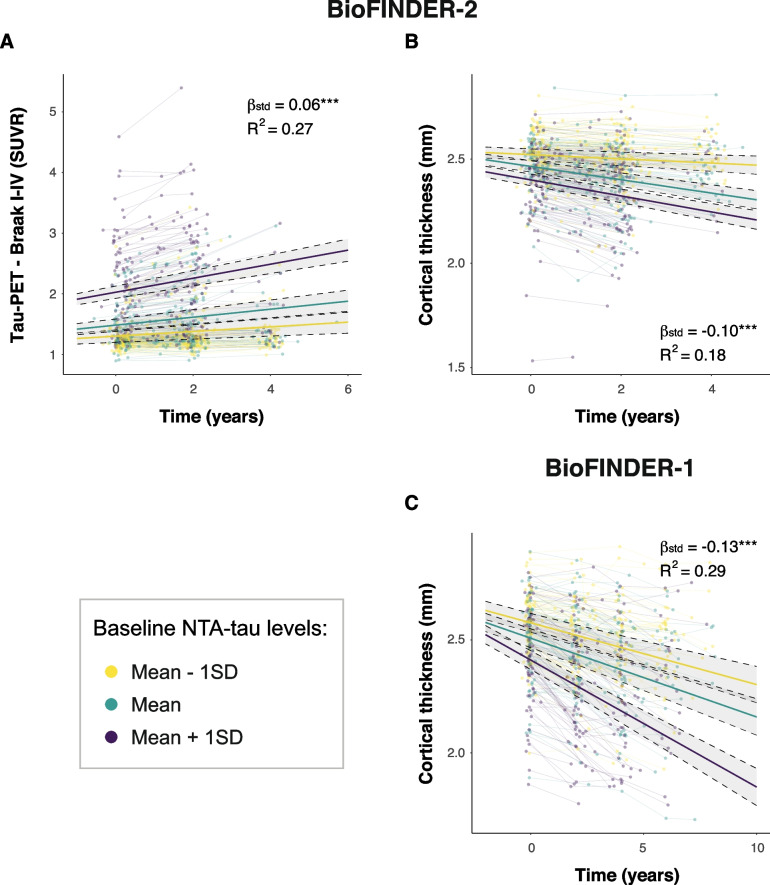
Baseline plasma NTA-tau association with longitudinal tau-PET and neurodegeneration. Associations between baseline plasma NTA-tau levels and longitudinal tau-PET (**A**) and cortical thickness determined through MRI (**B** and **C**, BioFINDER-2 and -1 respectively). We used linear mixed models with tau-PET (SUVR) and cortical thickness (mm) as outcome and the interaction of baseline plasma biomarkers and time as predictor with random intercepts and random time-slopes. Age and sex were used as covariates. Dots and thin lines represent individual timepoints and trajectories, respectively, for each participant. Each participant is coloured based on its baseline plasma NTA-tau levels. Thick lines and shaded areas represent the mean trajectory over time of each group of plasma NTA-tau baseline levels and its 95%CI. Standardized β (β_std_) and *p*-values of the associations as well as the R^2^ of the model are shown in the plots. Only Aβ+ within the AD *continuum* (excluding nonAD+) were included in these analyses, as were those expected to progress. Standardized β (β_std_) and *p*-values of the associations as well as the R^2^ of the model are shown in the plots. *: *p* < 0.05; **: *p* < 0.01; ***: *p* < 0.001. Abbreviations: Aβ, amyloid-β; AD, Alzheimer’s disease; CI, confidence interval; nonAD+ ; non-Alzheimer’s type dementia Aβ-positive; SUVR, standardized uptake value ratio

We assessed whether baseline plasma NTA-tau levels are useful in predicting longitudinal tau-PET increases, brain atrophy and cognitive decline in participants within the AD *continuum* (CU+ , MCI+ , AD+) at baseline (description in Supplementary Table 11–15, respectively). In BioFINDER-2, higher baseline levels of plasma NTA-tau were associated with higher longitudinal increases in tau-PET binding in the temporal meta-ROI (β[95%CI] = 0.06[0.05, 0.08], *p* < 0.001, R^2^ = 0.27, Fig. 5A and Table 3). We also observed that higher plasma NTA-tau levels at baseline were associated with a steeper decrease in cortical thickness in both BioFINDER-2 (β[95%CI] = -0.10[-0.13, -0.08], *p* < 0.001, R^2^ = 0.18; Fig. 5B) and BioFINDER-1 participants (β[95%CI] = -0.13[-0.16, -0.1], *p* < 0.001, R^2^ = 0.29; Fig. 5C).

**Table 3:** Associations between plasma NTA-tau and disease progression

NTA associations with:	*n*	β[95%CI]	p	R^2^
BioFINDER-2				
Tau-PET	294	0.06 [0.05, 0.08]	<0.001	0.27
Cortical thickness	288	-0.1 [-0.13, -0.08]	<0.001	0.18
MMSE	441	-1.04 [-1.27, -0.81]	<0.001	0.28
mPACC	380	-0.42 [-0.52, -0.32]	<0.001	0.27
BioFINDER-1				
Cortical thickness	212	-0.13 [-0.16, -0.1]	< 0.001	0.29
MMSE	324	-1.96 [-2.29, -1.63]	< 0.001	0.37
mPACC	211	-0.41 [-0.52, -0.31]	< 0.001	0.29

We used linear mixed models with tau-PET, cortical thickness or cognition (mPACC and MMSE) as outcome and the interaction of baseline plasma biomarkers and time as predictor with random intercepts and random time-slopes. Age and sex (and years of education for cognition) were used as covariates. Only Aβ+ within the AD *continuum* (excluding nonAD+) were included in these analyses, as were those expected to progress

*Abbreviations*: *Aβ* amyloid-β, *AD* Alzheimer’s disease, *MMSE* Mini-Mental State Examination, *nonAD+* non-Alzheimer’s type dementia Aβ positive, *mPACC* modified preclinical Alzheimer’s cognitive composite, *SUVR* standardized uptake value ratio

**Fig. 6:**
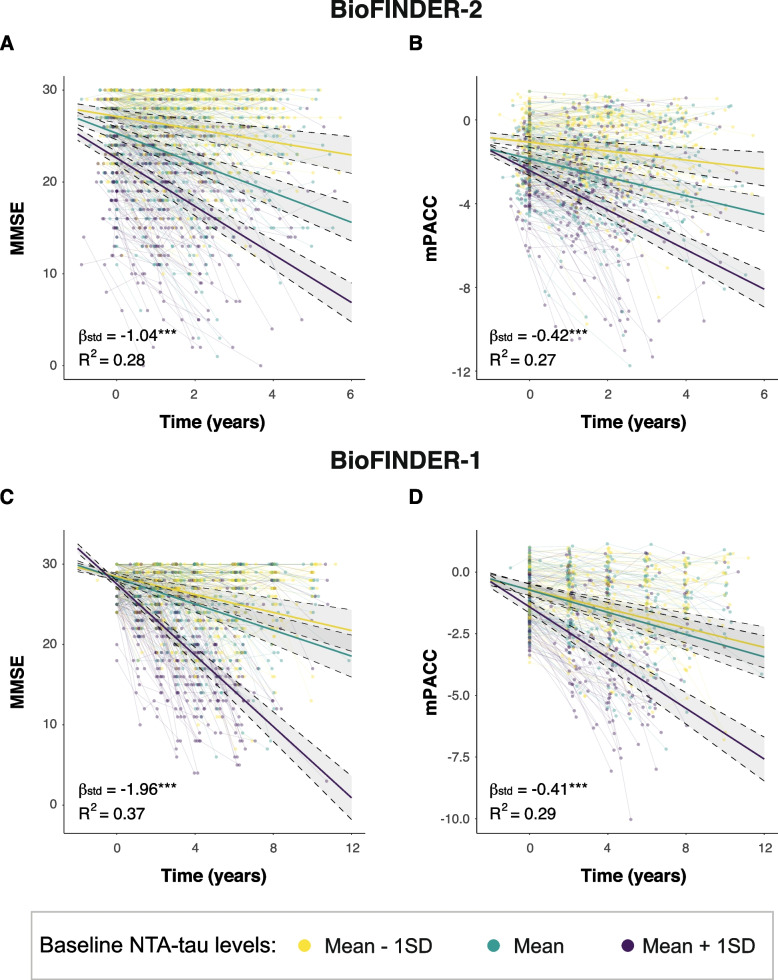
Baseline plasma NTA-tau association with cognitive decline. Associations between baseline plasma NTA-tau levels and longitudinal cognitive measures (**A** and **C**: MMSE, **B** and **D**: mPACC) in BioFINDER-2 (**A** and **B**) and BioFINDER-1 (**C** and **D**). We used linear mixed models with cognitive measures as outcome and the interaction of baseline plasma biomarkers and time as predictor with random intercepts and random time-slopes. Age, sex and years of education were used as covariates. Dots and thin lines represent individual timepoints and trajectories, respectively, for each participant. Each participant is coloured based on its baseline plasma NTA-tau levels. Thick lines and shaded areas represent the mean trajectory over time of each group of plasma NTA-tau baseline levels and its 95%CI. Only Aβ+ within the AD *continuum* (excluding nonAD+) were included in these analyses, as were those expected to progress. *: *p* < 0.05; **: *p* < 0.01; ***: *p* < 0.001. Abbreviations: Aβ, amyloid-β; AD, Alzheimer’s disease; CI, confidence interval; MMSE, Mini-Mental State Examination; mPACC, mPACC, modified preclinical Alzheimer’s cognitive composite; nonAD+ ; non-Alzheimer’s type dementia Aβ-positive

Next, we investigated whether baseline plasma NTA-tau concentrations can predict cognitive decline in participants across the AD *continuum* (CU+ , MCI+ , AD+). In BioFINDER-2, higher baseline plasma NTA-tau levels were associated with a more pronounced cognitive decline, both looking at MMSE (β[95%CI] = -1.04[-1.27, -0.81], *p* < 0.001, R^2^ = 0.28) and mPACC (β[95%CI] = -0.42[-0.52, -0.32], *p* < 0.001, *R*^2^ = 0.27) (Fig. 6A, B and Table 3). The same was observed in and BioFINDER-1: MMSE (β[95%CI] = -1.96, *p* < 0.001, R^2^ = 0.37) and mPACC (β[95%CI] = -0.41, *p* < 0.001, R^2^ = 0.29) (Fig. 6C, D and Table 3).

### Longitudinal changes in plasma NTA-tau

**Fig. 7:**
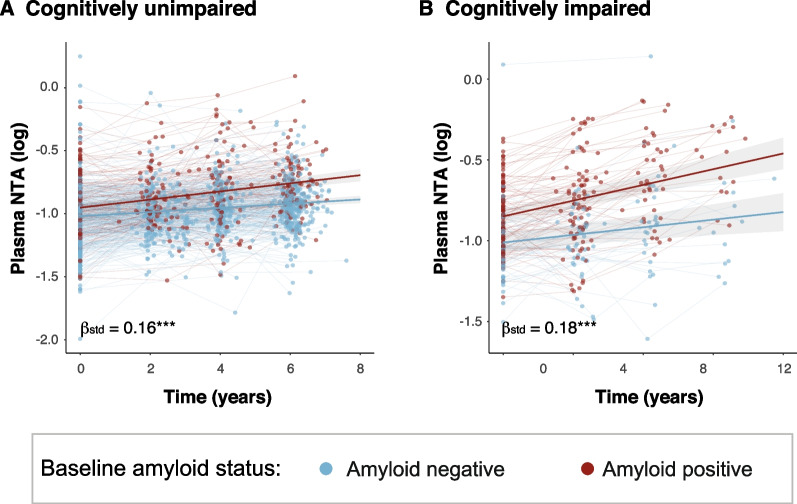
Longitudinal plasma NTA-tau association with baseline Aβ status. Longitudinal plasma NTA-tau changes classified by baseline Aβ status (negative, blue; positive, red) in in cognitively unimpaired (**A**) and impaired participants (**B**) from BioFINDER-1. Plasma NTA-tau levels were used as outcome in linear mixed models with the interaction between Aβstatus and time as predictor. Age and sex were included as covariates. Aβ-status was based on CSF Aβ42/40 levels. Standardized β (β_std_) and p-values of the interaction term are shown in the plots. *: *p* < 0.05; **: *p* < 0.01; ***: *p* < 0.001. Abbreviations: Aβ, amyloid-β; CSF, cerebrospinal fluid

Longitudinal measures of plasma NTA-tau were available in BioFINDER-1 participants (Supplementary Table 16). First, we investigated whether these changes were different by Aβ status at baseline. We found that plasma NTA-tau displayed higher longitudinal increases in Aβ pathology positive compared with negative cases both in CU (time × Aβ-interaction: β[95%CI] = 0.16[0.08, 0.25], *P* < 0.001; Fig. 7A) and CI participants (time × Aβ-interaction: β[95%CI] = 0.18[0.05, 0.31], *P* < 0.001; Fig. 7B).

**Fig. 8:**
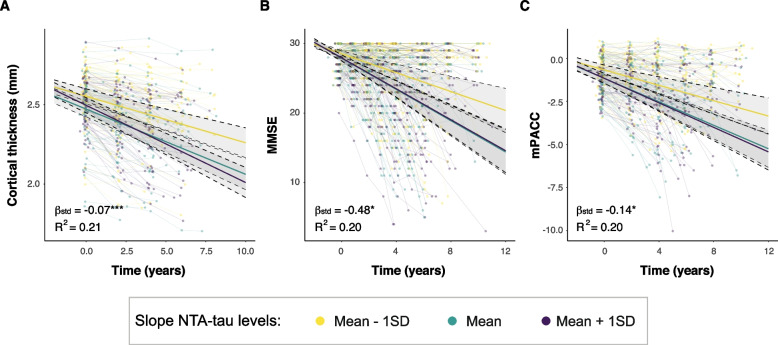
Longitudinal plasma NTA-tau association with over time changes in brain atrophy and cognition. Association between longitudinal plasma NTA-tau levels and longitudinal changes in cortical thickness (**A**) and cognitive performance (**B**, MMSE; **C**, mPACC). We used linear mixed models with cognitive measures as outcome and the interaction of plasma NTA-tau longitudinal changes and time as predictor with random intercepts and random time-slopes. Age, sex and education were used as covariates. Plasma NTA-tau longitudinal changes were derived from a linear mixed model with time as the only predictor, with random slopes and intercepts. Dots and thin lines represent individual timepoints and trajectories of cortical thickness or cognitive measures for each participant. Each participant is coloured based on its longitudinal plasma NTA-tau changes. Thick lines and shaded areas represent the mean trajectory over time of each group of plasma NTA-tau slopes and its 95%CI. Only Aβ+ within the AD *continuum* (excluding nonAD+) were included in these analyses, as were those expected to progress. *: *p* < 0.05; **: *p* < 0.01; ***: *p* < 0.001. Abbreviations: Aβ, amyloid-β; CI, confidence interval; MMSE, Mini-Mental State Examination; mPACC, mPACC, modified preclinical Alzheimer’s cognitive composite; nonAD+ ; non-Alzheimer’s type dementia Aβ-positive

Next, we investigated whether these longitudinal changes in plasma NTA-tau levels were related with disease progression. We observed that longitudinal changes in plasma NTA-tau were associated with over time changes in atrophy (β[95%CI] = -0.07 [-0.11, -0.03], *p* < 0.001, R^2^ = 0.21) in cognitive scores for both MMSE (β[95%CI] = -0.48[-0.87, -0.08], *p* < 0.05, R^2^ = 0.20) and mPACC (β[95%CI] = -0.14[-0.27, 0], *p* < 0.05, R^2^ = 0.20) (Fig. 8A-C).

## Discussion

In this study, we characterized a novel ultrasensitive immunoassay, referred to as NTA, capable of measuring N-terminal containing tau fragments (NTA-tau) in blood. For this purpose, we measured plasma NTA-tau levels in two large cohorts including cross-sectional and longitudinal samples of individuals across the AD *continuum*, healthy controls and nonAD cases, all well-characterized by clinically validated CSF and imaging biomarkers. Our main findings demonstrate that (i) plasma NTA-tau is increased across the AD *continuum,* abnormally emerging during preclinical AD stages, and showing the most prominent increases during late AD stages (AD+, A+T+, Braak V-VI, and A+MTL+N++), (ii) plasma NTA-tau levels in AD are strongly associated with in vivo deposition of insoluble tau aggregates as measured with tau-PET, (iii) NTA-tau levels in plasma are almost exclusively explained by in vivo tau aggregate pathology and (iv) plasma NTA-tau is capable of predicting disease progression (including tau accumulation, brain atrophy and cognitive decline) in participants within the AD *continuum* and (v) plasma NTA-tau changes are related with disease progression. Altogether, this suggests that plasma NTA-tau may have potential in clinical trials, as a pre-screening tool or as an outcome measure, and for patient management and monitoring in primary care.

Previous results with NTA-tau already showed promising increases in intermediate to late stages of the AD *continuum* [18]. For instance, in a clinical cohort, CSF NTA-tau was reported to be increased in MCI+ and AD+ compared with CSF Aβ negative cognitively impaired cases and control cases. Furthermore, in a small plasma pilot clinical cohort, preliminary results showed that NTA-tau concentrations were higher in AD compared with CU- and MCI- cases [18]. These findings were later corroborated in another study, where CSF NTA-tau was significantly higher in MCI+ and AD+ groups, whereas plasma NTA-tau was only increased in AD+ cases [44]. In the present study, we expanded these findings by demonstrating that plasma NTA-tau is increased across the AD *continuum*, starting to emerge subtly already at preclinical AD stages in asymptomatic cases, and showing that the bulk of the increase occurs between MCI+ and AD+ , with the latter group showing the highest concentrations among all investigated clinical groups. This suggests that AD pathophysiological changes occurring at later disease stages are responsible for the pronounced increase in plasma NTA-tau observed in dementia. This is further supported by the stratification of participants into AT groups, PET Braak stages, and Aβ- and tau-PET stages, where plasma NTA-tau concentrations were significantly and more prominently increased in A+T+ , Braak V-VI and A+MTL+N+ + groups. The pronounced increase observed in Braak V-VI cases was also observed previously [44], and the present results, together with the marked increase in A+MTL+N+ + participants, further corroborates preceding findings by highlighting the late nature of plasma NTA-tau as an AD biomarker. Similarly, the notably lower accuracy of plasma NTA-tau discriminating Aβ-PET compared with tau-PET indicates that the abnormal emergence of this biomarker across the AD *continuum* is closer in time to tau-PET crossing the positivity threshold, thus providing another indication of its late-stage nature. We also demonstrate that plasma NTA-tau is increased in Aβ-PET positive cases compared with the nonAD- group, however, the seemingly AD specificity of NTA-tau observed here should be interpreted with caution. In CSF, NTA-tau concentrations were significantly higher in Creutzfeldt-Jakob’s disease and acute neurological diseases (e.g. ischemic stroke) than in AD [18], and therefore it is feasible to hypothesize that the observed increases in CSF may translate also to plasma measurements in more acute neurological conditions, although none of these are expected to be confounded with AD’s clinical presentation. The ability of NTA assay to capture also non-phosphorylated tau species means that this biomarker is capable of detecting the marked intense neuronal damage and neurodegeneration of acute neurological conditions. This implies that the utility of plasma NTA-tau is likely to expand beyond AD, and therefore further studies exploring acute neurological conditions are warranted.

Among all recently developed blood tau biomarkers, p-tau species are especially promising and have contributed significantly to making ever closer the long-sought goal of blood testing for AD in memory clinics. However, accumulating evidence in recent years has challenged the idea of p-tau being purely a biomarker of insoluble tau aggregate pathology, calling into question its classification as a T biomarker within the AT(N) framework. First, p-tau species abnormally emerge early during preclinical AD stages, when tau-PET is still normal [33, 57, 58]. Thus, this shows that p-tau increases represent a relatively early event in the Aβ cascade [30, 33, 35, 59]. Further, it has also been demonstrated that some of these p-tau markers (*i.e.*, p-tau181, p-tau217 and p-tau231) may be equally or more tightly (depending on the epitope) associated with Aβ plaque pathology than tau tangle pathology, using both imaging biomarkers [60–62] and neuropathology confirmed samples [63, 64]. Hence, given the referred evidence, it is fair to propose that p-tau, either in CSF or blood, does not represent a strict surrogate biomarker of NFT accumulation in brain. Moreover, because these p-tau biomarkers are associated with the emergence of Aβ pathology, even from the earliest stages of preclinical AD [31, 33, 46, 65, 66], it is difficult to determine later in the disease course, what is the contribution of tau deposition to the overall p-tau signal in blood. This is especially true in symptomatic AD stages, where p-tau biomarkers show strong association with in vivo measurements of both Aβ plaques and NFT measured by PET [30, 35, 66]. Thus, while this makes p-tau markers highly relevant for early detection of AD, disease diagnosis and patient monitoring, it also makes them unsuitable for specifically tracking insoluble tau aggregate pathology in brain—this being of major significance as tangle deposition, unlike Aβ plaques, strongly associates with clinical symptoms and cognitive decline [67, 68]. Taken together, this highlights the urgent need for a fluid biomarker capable of specifically tracking tau pathology in vivo.

The results presented here, together with those previously reported [44], suggest that plasma NTA-tau can be such a biomarker. While we observed a significant association between plasma NTA-tau with Aβ-PET, tau-PET and neurodegeneration in Aβ-positive participants, our results demonstrate that plasma NTA-tau levels are strongly associated with tau pathology measured by tau-PET in Aβ-positive individuals, especially in regions known for the typical tau deposition in intermediate/late stages (Supplementary Fig. 2). This aligns well with previous findings, where plasma NTA-tau was shown to associate with amygdala, fusiform gyrus, parahippocampal gyrus, and lingual gyrus [44]. This, alongside its weak, yet significant, association with Aβ pathology, measured with CSF Aβ42/40, already suggests a very specific association with tau tangle pathology in AD. More importantly, when comparing plasma NTA-tau with plasma p-tau181, NfL and GFAP, it was NTA-tau which showed the strongest global and regional association with both tau-PET and neurodegeneration determined with MRI, while displaying comparatively weak association with Aβ-PET. Thus, this strongly suggests that, unlike any of the studied blood biomarkers, abnormal levels of plasma NTA-tau are indicative of underlaying tau pathology and associated brain atrophy. Moreover, thanks to the large amount of participants included in the BioFINDER-2 study, we can now fully confirm and expand previous findings, by showing that the variance in plasma NTA-tau levels is mainly associated with the tau-PET signal, even when adjusting for CSF Aβ42/40, which supports the claim of NTA-tau being specifically associated with insoluble tau deposition in AD. In this regard, we compared for the first time the contribution of Aβ (CSF Aβ42/40) and tau (tau-PET) on levels of other widely available plasma biomarkers (*i.e.*, p-tau181, GFAP and NfL). We showed that while all had a higher proportion of contribution from tau pathology, this was not significantly different from that of Aβ in most cases (*i.e.*, GFAP, NfL and t-tau). Only for plasma p-tau181, tau pathology measured by tau-PET had a significantly higher contribution than amyloid pathology measured by CSF Aβ42/40, but the contribution of the Aβ marker, in this case, was not negligible (Aβ = 18%, tau = 39.7%). On the other hand, for plasma NTA-tau the proportion of variation explained by CSF Aβ42/40 was very low (Aβ = 4.3%, tau = 52.9%). Altogether, these cross-sectional results suggest that NTA-tau may be a cost-effective and easily accessible alternative to tau-PET imaging, especially during intermediate and/or late stages of the disease. Moreover, and according to the NIA-AA Research Framework (which defines AD as a biological construct documented in vivo by biomarker evidence of both Aβ [A] and tau pathology [T]) [5], we propose that plasma NTA-tau may be a more suitable plasma T biomarker in AD than the ones currently used. Thus, these findings indicate the plasma NTA-tau could be a valuable tool for patient management in the clinical settings where it could serve as an easy to implement T biomarker, which may be especially important when disease-modifying treatments become widely available [69–72]. Additionally, plasma NTA-tau may be useful in clinical trials either as a pre-screening method or to assess the downstream effects of successful Aβ removal on tau deposition [73]. Finally, plasma NTA-tau could also be useful for selecting participants based on their tau pathology levels, as done in the donanemab trial with tau-PET [72].

Also supporting its utility, we found that plasma NTA-tau levels were associated with downstream measures of pathology. As expected by its relatively tight association with tau-PET [74, 75], we found that higher levels of NTA-tau was associated with neurodegeneration only in Aβ-positive participants, as estimated with lower cortical thickness, and with lower cognitive performance (determined both using MMSE and mPACC). Another novel contribution of this study is that higher levels of NTA-tau at baseline were associated with higher increases in tau-PET signal over time, increased atrophy, and steeper cognitive decline. As suggested by a recent paper, plasma biomarkers, such as plasma NTA-tau presented here, may be an easy way to increase the power of clinical trials by selecting those participants that have a higher risk of decline [76, 77]. In a previous publication, we showed that plasma NTA-tau concentrations increased longitudinally in MCI+ and AD+ cases, and that longitudinal changes plasma NTA-tau associated with tau-PET accumulation [44]. In this study, we further expanded these findings by demonstrating that baseline plasma NTA-tau levels were able to predict a higher risk of tau accumulation as detected by tau-PET, which may be especially useful for trials targeting tau pathology. Notably, we also demonstrated that high baseline plasma NTA-tau levels are predictive of steeper reduction in cortical thickness and steeper cognitive decline, which highlights the suitability of this novel plasma biomarker for tracking the down effects of AD pathophysiology and disease progression. Further supporting its clinical utility in AD, plasma NTA-tau showed significant amyloid pathology dependent changes over 8–10 years in both cognitively unimpaired and impaired AD cases. Moreover, longitudinal changes in plasma NTA-tau significantly associated with longitudinal changes in cognition and neurodegeneration over 12 years, highlighting the potential use of this novel biomarker. Altogether, these results support the use of plasma NTA-tau as a scalable, cost-effective and non-invasive substitute of tau-PET imaging also for prognosis, but also as a surrogate marker of disease progression (as evinced by its strong association with brain atrophy and cognitive decline), which may be useful both in the clinical settings and for clinical trials.

### Strengths and limitations

This study has several strengths that should be highlighted. First, the large sample size, including cases across the whole AD *continuum,* nonAD patients and healthy controls. Moreover, participants were very well-characterized, as they underwent detailed biochemical assessments (core AD CSF biomarkers) and various imaging examinations (including Aβ-PET, tau-PET and MRI). Further, a significant number of samples in the studied cohorts had available longitudinal data, which expanded for more than one decade in many participants. However, this study is not exempted of limitations. Aside from Quanterix plasma t-tau we did not have available t-tau measurements generated with other assays, thus we could not contextualize and compare NTA-tau with other t-tau biomarkers. Further, most of AD patients in BioFINDER-2 had no Aβ-PET available due to study design, which forced us to use CSF Aβ42/40 instead of Aβ-PET in some analyses. Finally, the lack of tau-PET imaging in BioFINDER-1 cohort did not allow us to further examine the association of plasma NTA-tau with insoluble tau deposition.

## Conclusions

Plasma NTA-tau is a biomarker that increases across the whole AD *continuum*, but it is especially elevated in the late disease stages. This is explained by its tight association with insoluble tau aggregate pathology, while its association with Aβ pathology is more limited. In our study, baseline plasma NTA-tau levels were also able to predict tau accumulation as measured with tau-PET, neurodegeneration determined by MRI cortical atrophy measures and cognitive decline. Moreover, longitudinal changes in plasma NTA-tau associated with amyloid pathology status in both preclinical and symptomatic cases, and also associated significantly with over time changes in brain atrophy and cognition. Overall, our results suggest that plasma NTA-tau could be used as a tau biomarker for AD diagnosis according to the AT(N) criteria and has potential for pre-screening and monitoring in clinical trials.

### Supplementary Information

**Additional file 1:** **Supplementary Figure 1.** Plasma NTA-tau levels across AA criteria for staging AD using PET (BioFINDER-2). **Supplementary Figure 2.** Regional associations between plasma NTA-tau, p-tau181, NfL and GFAP levels with Aβ-PET, tau-PET and cortical thickness (BioFINDER-2). **Supplementary Table 1.** Characteristics of the subsample with available plasma t-tau (BioFINDER-2). **Supplementary Table 2.** Plasma NTA-tau levels by diagnosis (BioFINDER-2). **Supplementary Table 3.** Characteristics of the sample by AT status (BioFINDER-2). **Supplementary Table 4.** Plasma NTA-tau levels by AT status (BioFINDER-2). **Supplementary Table 5.** Characteristics of the sample by Braak stages (BioFINDER-2). **Supplementary Table 6.** Plasma NTA-tau levels by Braak stages (BioFINDER-2). **Supplementary Table 7.** Plasma NTA-tau levels by AA criteria for staging AD (BioFINDER-2). **Supplementary Table 8.** Plasma NTA-tau levels by diagnosis (BioFINDER-1). **Supplementary Table 9.** Comparison between models including/excluding an interaction between plasma NTA-tau and Aβ-status (BioFINDER-2 and -1). **Supplementary Table 10.** Proportion of variation of plasma biomarker levels explained by amyloid and tau (BioFINDER-2). **Supplementary Table 11.** Characteristics of the longitudinal tau-PET sample (BioFINDER-2). **Supplementary Table 12.** Characteristics of the longitudinal MRI sample (BioFINDER-2). **Supplementary Table 13.** Characteristics of the longitudinal MRI sample (BioFINDER-1). **Supplementary Table 14.** Characteristics of the longitudinal cognition sample (BioFINDER-2). **Supplementary Table 15.** Characteristics of the longitudinal cognition sample (BioFINDER-1). **Supplementary Table 16.** Characteristics of the longitudinal plasma NTA-tau (BioFINDER-1).

## Data Availability

Anonymized data will be shared by request from a qualified academic investigator for the sole purpose of replicating procedures and results presented in the article and as long as data transfer is in agreement with EU legislation on the general data protection regulation and decisions by the Ethical Review Board of Sweden and Region Skåne, which should be regulated in a material transfer agreement.

## Abbreviations

Aβ: Amyloid-β
A-T-: Aβ and tau negative
A+T-: Aβ-positive tau negative
A+T+: Aβ and tau positive
A-T+: Aβ-negative tau positive
AD: Alzheimer’s disease
AD+: Alzheimer’s dementia Aβ-positive
ADAS-cog: Alzheimer’s Disease Assessment Scale-Cognitive subscale
AICc: Akaike information criteria
β_std_: Standardized β
CI: Confidence interval
CSF: Cerebrospinal fluid
CU: Cognitively unimpaired
CU-: Cognitively unimpaired Aβ-negative
CU+: Cognitively unimpaired Aβ-positive
FDR: False-discovery rate
GFAP: Glial fibrillary acidic protein
MCI: Mild cognitive impairment
MCI-: Mild cognitive impairment Aβ-negative
MCI+: Mild cognitive impairment Aβ-positive
MMSE: Mini-mental state examination
mPACC: Modified preclinical Alzheimer’s cognitive composite
MPRAGE: Magnetization-prepared rapid gradient echo
MRI: Magnetic resonance imaging
MTL: Medial temporal lobe
MTBR: Microtubule-binding region
N: Neocortical
NfL: Neurofilament light
NFTs: Neurofibrillary tangles
NIA-AA: National Institute of Aging and Alzheimer’s Association
NonAD-: Non-Alzheimer’s type dementia Aβ-negative
NonAD+: Non-Alzheimer’s type dementia Aβ-positive
NTA-tau: N-terminal tau containing fragments
PET: Positron emission tomography
P-tau: Phosphorylated tau
ROI: Region of interest
SUVR: Standardized uptake value
TIV: Total intracranial volume
TMT-A: Trail Making Test A
T-tau: Total tau
