# Supplementary material for: Plasma N-terminal containing tau fragments (NTA-tau): a biomarker of tau deposition in Alzheimer’s Disease
Source: Mol Neurodegener. 2024 Feb 17;19:19. doi: 10.1186/s13024-024-00707-x (PMC10874032; doi:10.1186/s13024-024-00707-x)
Supplement: Supplementary file 1 — Additional file 1: Supplementary Figure 1. Plasma NTA-tau levels across AA criteria for staging AD using PET (BioFINDER-2). Supplementary Figure 2. Regional associations between plasma NTA-tau, p-tau181, NfL and GFAP levels with Aβ-PET, tau-PET and cortical thickness (BioFINDER-2). Supplementary Table 1. Characteristics of the subsample with available plasma t-tau (BioFINDER-2). Supplementary Table 2. Plasma NTA-tau levels by diagnosis (BioFINDER-2). Supplementary Table 3. Characteristics of the sample by AT status (BioFINDER-2). Supplementary Table 4. Plasma NTA-tau levels by AT status (BioFINDER-2). Supplementary Table 5. Characteristics of the sample by Braak stages (BioFINDER-2). Supplementary Table 6. Plasma NTA-tau levels by Braak stages (BioFINDER-2). Supplementary Table 7. Plasma NTA-tau levels by AA criteria for staging AD (BioFINDER-2). Supplementary Table 8. Plasma NTA-tau levels by diagnosis (BioFINDER-1). Supplementary Table 9. Comparison between models including/excluding an interaction between plasma NTA-tau and Aβ-status (BioFINDER-2 and -1). Supplementary Table 10. Proportion of variation of plasma biomarker levels explained by amyloid and tau (BioFINDER-2). Supplementary Table 11. Characteristics of the longitudinal tau-PET sample (BioFINDER-2). Supplementary Table 12. Characteristics of the longitudinal MRI sample (BioFINDER-2). Supplementary Table 13. Characteristics of the longitudinal MRI sample (BioFINDER-1). Supplementary Table 14. Characteristics of the longitudinal cognition sample (BioFINDER-2). Supplementary Table 15. Characteristics of the longitudinal cognition sample (BioFINDER-1). Supplementary Table 16. Characteristics of the longitudinal plasma NTA-tau (BioFINDER-1). [file 13024_2024_707_MOESM1_ESM.docx]

**PLASMA N-TERMINAL CONTAINING TAU FRAGMENTS (NTA-TAU): A BIOMARKER OF TAU DEPOSITION IN ALZHEIMER’S DISEASE.**

Juan Lantero-Rodriguez^*^, Gemma Salvadó^*^, Anniina Snellman, Laia Montoliu-Gaya, Wagner S. Brum, Andrea L. Benedet, Niklas Mattsson-Carlgren, Pontus Tideman, Shorena Janelidze, Sebastian Palmqvist, Erik Stomrud, Nicholas J. Ashton, Henrik Zetterberg, Kaj Blennow, Oskar Hansson

**SUPPLEMENTARY FIGURES**

**Supplementary Figure 1: Plasma NTA-tau levels across AA criteria for staging AD using PET (BioFINDER-2)**

**Supplementary Figure 2: Regional associations between plasma NTA-tau, p-tau181, NfL and GFAP levels with Aβ-PET, tau-PET and cortical thickness (BioFINDER-2)**

**SUPPLEMENTARY TABLES**

**Supplementary Table 1: Characteristics of the subsample with available plasma t-tau (BioFINDER-2)**

**Supplementary Table 2: Plasma NTA-tau levels by diagnosis (BioFINDER-2)**

**Supplementary Table 3: Characteristics of the sample by AT status (BioFINDER-2)**

**Supplementary Table 4: Plasma NTA-tau levels by AT status (BioFINDER-2)**

**Supplementary Table 5: Characteristics of the sample by Braak stages (BioFINDER-2)**

**Supplementary Table 6: Plasma NTA-tau levels by Braak stages (BioFINDER-2)**

**Supplementary Table 7: Plasma NTA-tau levels by AA criteria for staging AD (BioFINDER-2)**

**Supplementary Table 8: Plasma NTA-tau levels by diagnosis (BioFINDER-1)**

**Supplementary Table 9: Comparison between models including/excluding an interaction between plasma NTA-tau and Aβ-status (BioFINDER-2 and -1)**

**Supplementary Table 10: Proportion of variation of plasma biomarker levels explained by amyloid and tau (BioFINDER-2)**

**Supplementary Table 11: Characteristics of the longitudinal tau-PET sample (BioFINDER-2)**

**Supplementary Table 12: Characteristics of the longitudinal MRI sample (BioFINDER-2)**

**Supplementary Table 13: Characteristics of the longitudinal MRI sample (BioFINDER-1)**

**Supplementary Table 14: Characteristics of the longitudinal cognition sample (BioFINDER-2)**

**Supplementary Table 15: Characteristics of the longitudinal cognition sample (BioFINDER-1)**

**Supplementary Table 16: Characteristics of the longitudinal plasma NTA-tau (BioFINDER-1)**

**SUPPLEMENTARY FIGURES**

**

**

**Supplementary Figure 1: Plasma NTA-tau levels across AA criteria for staging AD using PET (BioFINDER-2)**

Plasma NTA-tau levels in BioFINDER-2 by PET staging as defined in the new AA diagnostic criteria. Differences in plasma NTA-tau levels by groups were measured using ANCOVA and Tukey’s method for *post-hoc* comparisons. Age and sex were used as covariates in all cases. Aβ (A) status was assessed using Aβ-PET SUVR levels, when available, or CSF Aβ42/40 levels, medial temporal lobe (MTL) and neocortical (N) tau deposition was determined with tau-PET (based on previously validated cut-offs, for all). We divided the y-axis to show few cases with very high plasma NTA levels. Significance level *: p<0.05; **: p<0.01; ***: p<0.001. Box plots include all participants, displaying the median and the interquartile range; whiskers show the lower value of maximum/minimum value or 1.5 interquartile range from the hinge.

**

**

**Supplementary Figure 2: Regional associations between plasma NTA-tau, p-tau181, NfL and GFAP levels with Aβ-PET, tau-PET and cortical thickness (BioFINDER-2)**

Regional associations between plasma NTA-tau, p-tau181, NfL and GFAP levels with Aβ-PET (A), tau-PET (B) and cortical thickness (C) in Aβ-positive participants. Colours represent the standardized β (β_std_) only in areas with significant association (p_FDR_<0.05). For regional analyses, we investigated all FreeSurfer regions averaging the two hemispheres to reduce the number of comparisons. For subcortical regions we used volumes, instead of thickness, with the neurodegeneration-related analyses. Age and sex (and TIV for subcortical regions).

|  | All  (n=715) | CU-  (n=237) | CU+  (n=83) | MCI+ (n=87) | AD+  (n=102) | NonAD+ (n=48) | NonAD-(n=158) | p |
| --- | --- | --- | --- | --- | --- | --- | --- | --- |
| Age | 68.1 (12.3) | 60.2 (15.0) | 72.3 (8.6)*** | 72.4 (7.2)*** | 73.6 (6.6)*** | 74.8  (5.7)*** | 70.0  (9.4)*** | <0.001 |
| Women, n(%) | 350 (49.0%) | 127 (53.6%) | 41 (49.4%) | 44 (50.6%) | 58 (56.9%) | 20 (41.7%)*** | 60 (38.0%)*** | <0.001 |
| *APOE-ε4* carriers, n(%) | 351 (49.1%) | 80 (33.8%) | 60 (72.3%)*** | 67 (77.0%)*** | 75 (73.5%)*** | 32 (66.7%)*** | 37 (23.4%)*** | <0.001 |
| Education, years | 12.5 (3.9) | 12.7 (3.2) | 11.9 (3.7)* | 13.1 (4.9) | 12.4 (4.7) | 13.5  (4.1) | 11.9 (3.6)** | <0.001 |
| Imaging measures | | | | | | | |  |
| Centiloids^a^ | 15.4 (39.4) | -7.33 (8.53) | 45.0 (37.3)*** | 64.2 (43.3)*** | 96.1 (30.1)** | 68.4 (8.96)* | -5.71 (8.20) | <0.001 |
| Tau-PET SUVR^b^ | 1.34 (0.44) | 1.14 (0.10) | 1.25 (0.23)*** | 1.48 (0.42)*** | 2.12 (0.61)*** | 1.29 (0.19)*** | 1.15 (0.10) | <0.001 |
| Cortical thickness^c^ | 2.50 (0.16) | 2.59 (0.11) | 2.54 (0.12)** | 2.47 (0.11)*** | 2.32 (0.14)*** | 2.36 (0.16)*** | 2.51 (0.14)*** | <0.001 |
| Cognition | | | | | | | |  |
| MMSE | 26.4 (4.1) | 29.0 (1.2) | 28.8  (1.3) | 26.5 (1.9)*** | 20.0 (4.3)*** | 23.8 (5.1)*** | 26.1 (3.4)*** | <0.001 |
| mPACC^d^ | -1.29 (1.91) | 0.17 (0.70) | -0.34 (0.77)*** | -2.05 (0.97)*** | -4.13 (1.54)*** | -2.67 (2.33)*** | -1.84 (1.67)*** | <0.001 |
| Plasma levels | | | | | | | |  |
| Plasma NTA | 0.26 (0.16) | 0.21 (0.11) | 0.25 (0.12)** | 0.28 (0.13)*** | 0.46 (0.22)*** | 0.25  (0.13) | 0.20  (0.13) | <0.001 |
| Plasma t-tau | 1.70 (0.63) | 1.65 (0.59) | 1.59  (0.60) | 1.74  (0.63) | 1.94 (0.76)** | 1.66  (0.58) | 1.67  (0.59) | <0.001 |

**SUPPLEMENTARY TABLES**

**Supplementary Table 1: Characteristics of the subsample with available plasma t-tau (BioFINDER-2)**

Mean (SD) is reported unless otherwise indicated. ^a^ 273 participants missing; ^b^ 31 participants missing; ^c^ 20 participants missing; ^d^ 53 participants missing. Abbreviations: Aβ, amyloid-β; AD+, Alzheimer’s dementia amyloid positive; A-T-, amyloid and tau negative; A+T-, amyloid positive tau negative; A+T+, amyloid and tau positive; A-T+, amyloid negative tau positive; CU-, cognitively unimpaired amyloid negative; CU+, cognitively unimpaired amyloid positive; MCI+, mild cognitive impairment amyloid positive; MMSE, Mini-Mental State Examination; non-AD+; non-Alzheimer’s type dementia amyloid positive; non-AD-, non-Alzheimer’s type dementia amyloid negative; mPACC, modified preclinical Alzheimer’s cognitive composite; SUVR, standardized uptake value ratio; t-tau, total tau.

|  | Cu- | CU+ | MCI+ | ADD+ | nonAD+ |
| --- | --- | --- | --- | --- | --- |
| Cu+ | **1.30 (<0.001)** | - | - | - | - |
| MCI+ | **1.39 (<0.001)** | 1.07 (0.870) | - | - | - |
| AD+ | **2.17 (<0.001)** | **1.67 (<0.001)** | **1.56 (<0.001)** | - | - |
| nonAD+ | 1.17 (0.680) | 0.90 (0.665) | 0.84 (0.159) | **0.54 (<0.001)** | - |
| nonAD- | 0.99 (0.993) | **0.77 (<0.001)** | **0.71 (<0.001)** | **0.46 (<0.001)** | 0.85 (0.461) |

**Supplementary Table 2: Plasma NTA-tau levels by diagnosis (BioFINDER-2)**

Differences of plasma levels by diagnostic groups were measured using ANCOVA and Tukey’s method for *post-hoc* comparisons. Size effects are shown in the table calculated as fold increases (FI). P-values of each comparison are shown between brackets. Significant differences (p<0.05) are shown in bold. Abbreviations: AD+, Alzheimer’s dementia amyloid positive; CU-, cognitively unimpaired amyloid negative; CU+, cognitively unimpaired amyloid positive; FI, fold increase; MCI+, mild cognitive impairment amyloid positive non-AD+; non-Alzheimer’s type dementia amyloid positive; non-AD-, non-Alzheimer’s type dementia amyloid negative.

|  | A-T-  (n=640) | A+T-  (n=254) | A+T+  (n=271) | A-T+  (n=17) | p-value |
| --- | --- | --- | --- | --- | --- |
| Age | 63.5 (14.1) | 72.4 (8.32)*** | 73.0 (7.10)*** | 70.4 (14.9)* | <0.001 |
| Women, n(%) | 311 (48.6%) | 114 (44.9%)*** | 140 (51.7%)*** | 8 (47.1%) | <0.001 |
| *APOE-ε4* carriers, n(%)^a^ | 206 (32.2%) | 159 (62.6%)*** | 194 (71.6%)*** | 2 (11.8%)*** | <0.001 |
| Education, years^b^ | 12.6 (3.34) | 12.5 (3.89) | 12.7 (4.41) | 12.7 (3.00) | 1 |
| Imaging measures |  |  |  |  |  |
| Centiloids^c^ | -7.17 (7.93) | 41.1 (36.0)*** | 89.1 (30.2)*** | -1.46 (8.88)** | <0.001 |
| Tau-PET SUVR | 1.14 (0.08) | 1.18 (0.09)*** | 1.98 (0.56)*** | 1.37 (0.06)*** | <0.001 |
| Cortical thickness^d^ | 2.57 (0.123) | 2.49 (0.149)*** | 2.39 (0.139)*** | 2.53 (0.113) | <0.001 |
| Cognition |  |  |  |  |  |
| MMSE^e^ | 28.1 (2.43) | 26.8 (3.67)*** | 23.2 (4.96)*** | 27.8 (2.99) | <0.001 |
| mPACC^f^ | -0.420 (1.38) | -1.33 (1.69)*** | -2.98 (1.99)*** | -0.676 (1.20) | <0.001 |
| Plasma levels |  |  |  |  |  |
| Plasma NTA | 0.204 (0.115) | 0.237 (0.130)*** | 0.403 (0.196)*** | 0.228 (0.153) | <0.001 |

**Supplementary Table 3: Characteristics of the sample by AT status (BioFINDER-2)**

Mean (SD) is reported unless otherwise indicated. P-values show differences among groups as calculated with Kruskal-Wallis or Chi-squared tests. *Post-hoc* analyses against A-T- group are shown in the cells. Amyloid (A) status was assessed using CSF Aβ42/40 levels and tau (T) status using tau-PET SUVR based on previously validated cut-offs. *: p<0.05; **: p<0.01; ***: p<0.001. Mean (SD) is reported unless otherwise indicated. ^a^ 43 participants missing; ^b^ 7 participants missing; ^c^ 140 participants missing; ^d^ 6 participants missing; ^d^ 2 participants missing; ^d^ 19 participants missing.

Abbreviations: Aβ, amyloid-β; A-T-, amyloid and tau negative; A+T-, amyloid positive tau negative; A+T+, amyloid and tau positive; A-T+, amyloid negative tau positive; MMSE, Mini-Mental State Examination; mPACC, modified preclinical Alzheimer’s cognitive composite; SUVR, standardized uptake value ratio.

|  | A-T- | A+T- | A+T+ |
| --- | --- | --- | --- |
| A+T- | 1.16 (0.022) | - | - |
| A+T+ | 1.98 (<0.001) | 1.70 (<0.001) | - |
| A-T+ | 1.12 (0.914) | 0.96 (0.994) | 0.56 (<0.001) |

**Supplementary Table 4: Plasma NTA-tau levels by AT status (BioFINDER-2)**

Differences of plasma levels by diagnostic groups were measured using ANCOVA and Tukey’s method for *post-hoc* comparisons. Size effects are shown in the table calculated as fold increases (FI). P-values of each comparison are shown between brackets. Significant differences (p<0.05) are shown in bold. Amyloid (A) status was assessed using CSF Aβ42/40 levels and tau (T) status using tau-PET SUVR based on previously validated cut-offs.

Abbreviations: Aβ, amyloid-β; A-T-, amyloid and tau negative; A+T-, amyloid positive tau negative; A+T+, amyloid and tau positive; A-T+, amyloid negative tau positive; FI, fold increase.

|  | Braak 0  (n=785) | Braak I-II  (n=100) | Braak III-IV  (n=72) | Braak V-VI  (n=181) | p-value |
| --- | --- | --- | --- | --- | --- |
| Age | 65.1 (13.3) | 75.4 (6.83)*** | 75.1 (6.82)*** | 71.6 (8.16)*** | <0.001 |
| Women, n(%) | 367 (46.8%) | 56 (56.0%) | 32 (44.4%) | 100 (55.2%) | <0.001 |
| *APOE-ε4* carriers, n(%)^a^ | 305 (38.9%) | 58 (58.0%) | 50 (69.4%) | 133 (73.5%) | <0.001 |
| Education, years^b^ | 12.6 (3.47) | 12.1 (3.80) | 13.1 (4.73) | 12.5 (4.23) | <0.001 |
| Imaging measures |  |  |  |  |  |
| Centiloids^c^ | 1.54 (23.6) | 53.2 (43.5)*** | 84.4 (35.4)*** | 86.8 (37.7)*** | <0.001 |
| Tau-PET SUVR | 1.13 (0.08) | 1.26 (0.05)*** | 1.49 (0.15)*** | 2.22 (0.50)*** | <0.001 |
| Cortical thickness^d^ | 2.55 (0.135) | 2.51 (0.129)** | 2.44 (0.137)*** | 2.37 (0.140)*** | <0.001 |
| Cognition |  |  |  |  |  |
| MMSE^e^ | 28.0 (2.70) | 26.2 (3.78)*** | 25.3 (4.55)*** | 22.3 (4.96)*** | <0.001 |
| mPACC^f^ | -0.557 (1.48) | -1.73 (1.55)*** | -2.03 (1.42)*** | -3.38 (2.09)*** | <0.001 |
| Plasma levels |  |  |  |  |  |
| Plasma NTA-tau | 0.209 (0.117) | 0.260 (0.153)** | 0.310 (0.154)*** | 0.445 (0.195)*** | <0.001 |

**Supplementary Table 5: Characteristics of the sample by Braak stages (BioFINDER-2)**

Mean (SD) is reported unless otherwise indicated. P-values show differences among groups as calculated with Kruskal-Wallis or Chi-squared tests. *Post-hoc* analyses against Braak 0 group are shown in the cells. Participants with available tau-PET imaging were stratified according to the PET Braak stages into: Braak 0, Braak I-II, Braak III-IV, and Braak V-VI in a hierarchical manner, based on regional SUVR cut-offs (Braak I-II:1.38, Braak III-IV: 1.32, Braak V-VI: 1.19). Mean (SD) is reported unless otherwise indicated. ^a^ 53 participants missing; ^b^ 14 participants missing; ^c^ 173 participants missing; ^d^ 7 participants missing; ^e^ 2 participants missing; ^f^ 26 participants missing.

*: p<0.05; **: p<0.01; ***: p<0.001.

Abbreviations: MMSE, Mini-Mental State Examination; mPACC, modified preclinical Alzheimer’s cognitive composite; SUVR, standardized uptake value ratio.

|  | Braak 0 | Braak I-II | Braak III-IV |
| --- | --- | --- | --- |
| Braak I-II | 1.24 (0.016) | - | - |
| Braak III-IV | 1.48 (<0.001) | 1.19 (0.088) | - |
| Braak V-VI | 2.13 (<0.001) | 1.71 (<0.001) | 1.44 (<0.001) |

**Supplementary Table 6: Plasma NTA-tau levels by Braak stages (BioFINDER-2)**

Differences of plasma levels by diagnostic groups were measured using ANCOVA and Tukey’s method for *post-hoc* comparisons. Size effects are shown in the table calculated as fold increases (FI). P-values of each comparison are shown between brackets. Significant differences (p<0.05) are shown in bold. Participants with available tau-PET imaging were stratified according to the PET Braak stages into: Braak 0, Braak I-II, Braak III-IV, and Braak V-VI in a hierarchical manner, based on regional SUVR cut-offs (Braak I-II:1.38, Braak III-IV: 1.32, Braak V-VI: 1.19).

Abbreviations: FI, fold increase.

|  | A-MTL-N- | A+MTL-N- | A+MTL+N- | A+MTL+N+ |
| --- | --- | --- | --- | --- |
| A+MTL-N- | 0.86 (0.765) | **-** |  |  |
| A+MTL+N- | 1.58 (<0.001) | 1.83 (<0.001) | **-** |  |
| A+MTL+N+ | 1.63 (<0.001) | 1.89 (<0.001) | 1.03 (0.997) | **-** |
| A+MTL+N++ | 2.59 (<0.001) | 3.01 (<0.001) | 1.64 (<0.001) | 1.59 (<0.001) |

**Supplementary Table 7: Plasma NTA-tau levels by PET stages (BioFINDER-2)**

Differences of plasma levels by PET stages based on the AA diagnostic criteria were measured using ANCOVA and Tukey’s method for *post-hoc* comparisons. Size effects are shown in the table calculated as fold increases (FI). P-values of each comparison are shown between brackets. Significant differences (p<0.05) are shown in bold. Aβ (A) status was assessed using Aβ-PET SUVR levels, when available, or CSF Aβ42/40 levels, medial temporal lobe (MTL) and neocortical (N) tau deposition was determined with tau-PET (based on previously validated cut-offs, for all). Abbreviations: FI, fold increase.

|  | CU- | CU+ | MCI+ |
| --- | --- | --- | --- |
| CU+ | 1.23 (0.071) |  |  |
| MCI+ | 1.65 (<0.001) | 1.35 (<0.001) |  |
| nonAD- | 1.03 (0.974) | 0.84 (0.62) | 0.62 (<0.001) |

**Supplementary Table 8: Plasma NTA-tau levels by diagnosis (BioFINDER-1)**

Differences of plasma levels by diagnostic groups were measured using ANCOVA and Tukey’s method for *post-hoc* comparisons. Size effects are shown in the table calculated as fold increases (FI). P-values of each comparison are shown between brackets. Significant differences (p<0.05) are shown in bold. Abbreviations: AD+, Alzheimer’s dementia amyloid positive; CU-, cognitively unimpaired amyloid negative; CU+, cognitively unimpaired amyloid positive; FI, fold increase; MCI+, mild cognitive impairment amyloid positive; non-AD-, non-Alzheimer’s type dementia amyloid negative.

|  | Without interaction | | With interaction | | Comparison | |
| --- | --- | --- | --- | --- | --- | --- |
| Biomarkers | AICc | R^2^ | AICc | R^2^ | F-test | p |
| BioFINDER-2 |  |  |  |  |  |  |
| Aβ-PET | 1543.3 | 0.64 | 1505.5 | 0.65 | 40.5 | <0.001 |
| Tau-PET | 2734.1 | 0.41 | 2580.0 | 0.48 | 166.2 | <0.001 |
| Cortical thickness | 2241.1 | 0.37 | 2257.6 | 0.36 | 18.6 | <0.001 |
| BioFINDER-1 |  | | | | | |
| Aβ-PET | 0.70 | 357.3 | 0.72 | 345.2 | 14.3 | <0.001 |
| Cortical thickness | 0.18 | 1778.2 | 0.20 | 1763 | 17.3 | 0 |

**Supplementary Table 9: Comparison between models including/excluding an interaction between plasma NTA-tau and Aβ-status (BioFINDER-2 and -1)**

Linear regression models were used to assess the association between Aβ, tau or neurodegeneration and plasma levels in independent models with age and sex as covariates. We compared models including/excluding an interaction between plasma NTA-tau and Aβ-status. Non-AD participants were excluded in the cortical thickness analyses, to avoid bias. Comparisons were performed with an F-test. Significant differences (p<0.05) support the use of the model with the interaction between plasma NTA-tau and Aβ-status.

Abbreviations: Aβ, amyloid-β; AICc, corrected Akaike information criteria; nonAD; non-Alzheimer’s type dementia.

|  | pR^2^ Aβ | ppR^2^ Aβ | pR^2^ tau | ppR^2^ tau | R^2^ | ΔR^2^ | p |
| --- | --- | --- | --- | --- | --- | --- | --- |
| NTA | 0.01 | 4.3% | 0.15 | 52.9% | 0.28 | 0.14 | **<0.001** |
| p-tau181 | 0.08 | 18% | 0.17 | 39.7% | 0.43 | 0.09 | **0.020** |
| GFAP | 0.03 | 4.8% | 0.07 | 12.9% | 0.54 | 0.04 | 0.109 |
| NfL | 0.01 | 1.4% | 0.02 | 3.2% | 0.61 | 0.01 | 0.494 |
| NTA | 0.01 | 4.4% | 0.16 | 54.4% | 0.30 | 0.15 | **<0.001** |
| t-tau | 0.00 | 7.2% | 0.01 | 25.6% | 0.06 | 0.01 | 0.454 |

**Supplementary Table 10: Proportion of variation of plasma biomarker levels explained by amyloid and tau (BioFINDER-2)**

Proportion of variation of plasma biomarker levels explained by amyloid and tau was calculated by partial R^2^ (pR^2^) in multivariable linear regression models with amyloid (CSF Aβ42/40) and tau (tau-PET, log-transformed) as predictors and each plasma biomarker (log-transformed) as outcome, adjusting for age and sex. Percentual partial R^2^ (ppR^2^) were calculated as the partial R^2^ of each predictor divided by the total R^2^ of the model (100 * pR^2^/R^2^). ΔR^2^ represents the difference between amyloid and tau partial R2, with positive values meaning more proportion of variance explained by tau (pR^2^ tau - pR^2^ Aβ). Differences on partial R^2^ were assessed by bootstrapping, and significant p-values (p<0.05, in bold) represent significant differences on the contribution of tau and amyloid pathologies on each of the plasma biomarker levels.

|  | All (n=210) | CU+ (n=80) | MCI+ (n=65) | AD+ (n=65) |
| --- | --- | --- | --- | --- |
| Age | 72.0 (7.8) | 71.5 (8.8) | 72.1 (7.5) | 72.6 (6.9) |
| Women, n(%) | 108 (51.4%) | 45 (56.3%) | 29 (44.6%) | 34 (52.3%) |
| *APOE-ε4* carriers, n(%) | 149 (71.0%) | 58 (72.5%) | 47 (72.3%) | 44 (67.7%) |
| Education years^a^ | 12.2 (4.2) | 11.9 (3.7) | 13.0 (4.8) | 11.8 (3.9) |
| Tau-PET SUVR | 1.61 (0.59) | 1.26 (0.24) | 1.49 (0.43) | 2.15 (0.64) |
| Time follow-up, years, [min, max] | 2.02 (0.73)  [0.731, 4.13] | 2.28 (0.86)  [1.23, 4.13] | 2.04 (0.74)  [0.851, 3.85] | 1.68 (0.33)  [0.731, 2.46] |
| Number of visits,  [min, max] | 2.40 (0.60)  [2.00, 4.00] | 2.45 (0.65)  [2.00, 4.00] | 2.46 (0.64)  [2.00, 4.00] | 2.26 (0.44)  [2.00, 3.00] |

**Supplementary Table 11: Characteristics of the longitudinal tau-PET sample (BioFINDER-2)**

Mean (SD) is reported unless otherwise indicated. ^a^ 2 participants missing.

Abbreviations: AD+, Alzheimer’s dementia amyloid positive; CU+, cognitively unimpaired amyloid positive; MCI+, mild cognitive impairment amyloid positive; SUVR, standardized uptake value ratio.

|  | All (n=288) | CU+ (n=109) | MCI+ (n=89) | AD+ (n=90) |
| --- | --- | --- | --- | --- |
| Age | 71.4 (7.64) | 69.9 (8.67) | 72.0 (6.46) | 72.7 (7.17) |
| Women, n(%) | 146 (50.7%) | 59 (54.1%) | 38 (42.7%) | 49 (54.4%) |
| *APOE-ε4* carriers, n(%) | 209 (72.6%) | 81 (74.3%) | 67 (75.3%) | 61 (67.8%) |
| Education years^a^ | 12.6 (4.26) | 12.8 (3.82) | 12.9 (4.60) | 12.0 (4.43) |
| Plasma NTA-tau | 0.327 (0.191) | 0.250 (0.134) | 0.326 (0.247) | 0.635 (1.54) |
| Tau-PET SUVR | 2.46 (0.152) | 2.54 (0.126) | 2.47 (0.104) | 2.35 (0.156) |
| Time follow-up, years, | 1.22 (1.21) | 1.32 (1.33) | 1.44 (1.29) | 0.884 (0.859) |

**Supplementary Table 12: Characteristics of the longitudinal MRI sample (BioFINDER-2)**

Mean (SD) is reported unless otherwise indicated. ^a^ 9 participants missing.

Abbreviations: AD+, Alzheimer’s dementia amyloid positive; CU+, cognitively unimpaired amyloid positive; MCI+, mild cognitive impairment amyloid positive; SUVR, standardized uptake value ratio.

|  | All (n=212) | CU+ (n=118) | MCI+ (n=94) |
| --- | --- | --- | --- |
| Age | 72.9 (5.10) | 73.5 (5.20) | 72.1 (4.88) |
| Women, n(%) | 110 (51.9%) | 69 (58.5%) | 41 (43.6%) |
| *APOE-ε4* carriers, n(%) | 140 (66.0%) | 72 (61.0%) | 68 (72.3%) |
| Education years^a^ | 11.9 (3.57) | 12.1 (3.73) | 11.6 (3.35) |
| Plasma NTA-tau | 0.151 (0.0986) | 0.137 (0.101) | 0.168 (0.0937) |
| Cortical thickness (mm) | 2.34 (0.242) | 2.40 (0.246) | 2.26 (0.214) |
| Time follow-up, years, | 4.36 (1.79) | 4.85 (1.80) | 3.75 (1.58) |

**Supplementary Table 13: Characteristics of the longitudinal MRI sample (BioFINDER-1)**

Mean (SD) is reported unless otherwise indicated. ^a^ 1 participants missing.

Abbreviations: AD+, Alzheimer’s dementia amyloid positive; CU+, cognitively unimpaired amyloid positive; MCI+, mild cognitive impairment amyloid positive; SUVR, standardized uptake value ratio.

|  | All (n=318) | CU+ (n=102) | MCI+ (n=101) | AD+ (n=115) |
| --- | --- | --- | --- | --- |
| Age | 72.2 (7.7) | 70.9 (8.8) | 72.1 (7.4) | 73.3 (6.7) |
| Women, n(%) | 161 (50.6%) | 52 (51.0%) | 49 (48.5%) | 60 (52.2%) |
| *APOE-ε4* carriers, n(%) | 230 (72.3%) | 72 (70.6%) | 75 (74.3%) | 83 (72.2%) |
| Education years | 12.4 (4.20) | 12.1 (3.53) | 13.0 (4.71) | 12.3 (4.26) |
| MMSE | 25.1 (4.7) | 28.7 (1.4) | 26.6 (1.9) | 20.5 (4.5) |
| mPACC^a^ | -2.10 (1.94) | -0.288 (0.76) | -2.00 (0.93) | -4.06 (1.68) |
| Time follow-up, years, [min, max] | 2.45 (0.88) [0.402, 4.50] | 2.81 (0.90) [0.402, 4.39] | 2.59 (0.92) [0.632, 4.50] | 1.99 (0.59) [0.479, 3.26] |
| Number of visits,  [min, max] | 3.01 (0.848) [2.00, 5.00] | 3.15 (1.01)  [2.00, 5.00] | 3.23 (0.92)  [2.00, 5.00] | 2.70 (0.46)  [2.00, 3.00] |

**Supplementary Table 14: Characteristics of the longitudinal cognition sample (BioFINDER-2)**

Mean (SD) is reported unless otherwise indicated. ^a^ 28 participants missing.

Abbreviations: AD+, Alzheimer’s dementia amyloid positive; CU+, cognitively unimpaired amyloid positive; MCI+, mild cognitive impairment amyloid positive; MMSE, Mini Mental State Examination; mPACC, Preclinical Alzheimer’s Cognitive Composite; SUVR, standardized uptake value ratio.

|  | All (n=322) | CU+ (n=175) | MCI+ (n=147) |
| --- | --- | --- | --- |
| Age | 72.9 (5.17) | 73.1 (5.30) | 72.7 (5.02) |
| Women, n(%) | 175 (54.3%) | 107 (61.1%) | 68 (46.3%) |
| *APOE-ε4* carriers, n(%) | 189 (58.7%) | 87 (49.7%) | 102 (69.4%) |
| Education years | 11.6 (3.63) | 12.0 (3.78) | 11.1 (3.39) |
| MMSE | 27.7 (1.92) | 28.6 (1.43) | 26.6 (1.83) |
| mPACC^a^ | -1.28 (1.24) | -0.481 (0.839) | -2.14 (1.02) |
| Plasma NTA-tau | 0.160 (0.128) | 0.135 (0.0905) | 0.191 (0.157) |
| Time follow-up, years | 6.10 (2.51) | 6.85 (2.46) | 5.20 (2.27) |

**Supplementary Table 15: Characteristics of the longitudinal cognition sample (BioFINDER-1)**

Mean (SD) is reported unless otherwise indicated. ^a^ 50 participants missing.

Abbreviations: CU+, cognitively unimpaired amyloid positive; MCI+, mild cognitive impairment amyloid positive; MMSE, Mini Mental State Examination; mPACC, Preclinical Alzheimer’s Cognitive Composite; SUVR, standardized uptake value ratio.

|  | All (n=231) | CU+ (n=145) | MCI+ (n=86) |
| --- | --- | --- | --- |
| Age | 72.6 (5.01) | 73.1 (5.00) | 71.7 (4.93) |
| Women, n(%) | 132 (57.1%) | 88 (60.7%) | 44 (51.2%) |
| *APOE-ε4* carriers, n(%)^a^ | 131 (56.7%) | 68 (46.9%) | 63 (73.3%) |
| Education years^b^ | 11.7 (3.50) | 11.9 (3.61) | 11.4 (3.30) |
| MMSE | 27.9 (1.82) | 28.6 (1.48) | 26.7 (1.70) |
| mPACC^c^ | -1.13 (1.17) | -0.498 (0.864) | -2.05 (0.912) |
| Plasma NTA-tau | 0.213 (0.766) | 0.242 (0.965) | 0.164 (0.0828) |
| Time follow-up, years | 6.52 (2.13) | 7.01 (2.09) | 5.70 (1.96) |

**Supplementary Table 16: Characteristics of the longitudinal plasma NTA-tau (BioFINDER-1)**

Mean (SD) is reported unless otherwise indicated. ^a^ 31 participants missing; ^b^ 1 participants missing; ^c^ 41 participants missing.

Abbreviations: CU+, cognitively unimpaired amyloid positive; MCI+, mild cognitive impairment amyloid positive; MMSE, Mini Mental State Examination; mPACC, Preclinical Alzheimer’s Cognitive Composite; SUVR, standardized uptake value ratio.
